# Energy balance drives diurnal and nocturnal brain transcriptome rhythms

**DOI:** 10.1016/j.celrep.2024.113951

**Published:** 2024-03-19

**Authors:** Laura van Rosmalen, Shaunak Deota, Geraldine Maier, Hiep D. Le, Terry Lin, Ramesh K. Ramasamy, Roelof A. Hut, Satchidananda Panda

**Affiliations:** 1 Regulatory Biology Laboratory, The Salk Institute for Biological Studies, 10010 North Torrey Pines Road, La Jolla, CA 92037, USA; 2 Chronobiology Unit, Groningen Institute for Evolutionary Life Sciences, University of Groningen, 9700 CC Groningen, the Netherlands; 3 Lead contact

## Abstract

Plasticity in daily timing of activity has been observed in many species, yet the underlying mechanisms driving nocturnality and diurnality are unknown. By regulating how much wheel-running activity will be rewarded with a food pellet, we can manipulate energy balance and switch mice to be nocturnal or diurnal. Here, we present the rhythmic transcriptome of 21 tissues, including 17 brain regions, sampled every 4 h over a 24-h period from nocturnal and diurnal male CBA/CaJ mice. Rhythmic gene expression across tissues comprised different sets of genes with minimal overlap between nocturnal and diurnal mice. We show that non-clock genes in the suprachiasmatic nucleus (SCN) change, and the habenula was most affected. Our results indicate that adaptive flexibility in daily timing of behavior is supported by gene expression dynamics in many tissues and brain regions, especially in the habenula, which suggests a crucial role for the observed nocturnal-diurnal switch.

## In brief

van Rosmalen et al. present a rhythmic transcriptome atlas of 21 tissues, including 17 brain regions, from nocturnal and diurnal mice. A distinct set of rhythmic genes is linked to the temporal niche. The habenula emerged as the most affected tissue, suggesting a role in the observed nocturnal-diurnal switch.

## Graphical abstract

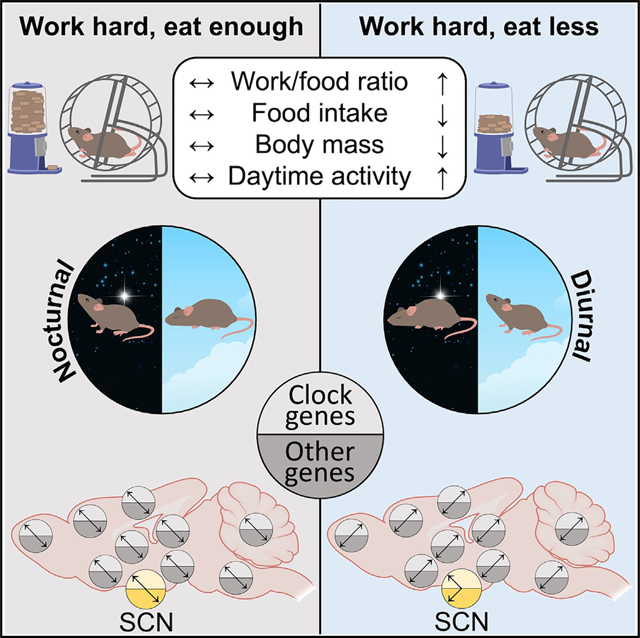

## INTRODUCTION

The suprachiasmatic nucleus (SCN) is the central clock controlling circadian rhythms in both diurnal and nocturnal species.^1^ However, the molecular and cellular networks responsible for the reversed circadian rhythms in behavior, physiology, and metabolism across diurnal and nocturnal mammals remain to be discovered. Brain regions that mediate circadian behavior are influenced by the clock.^2,3^ Genes within these neuronal networks may contribute to the daily rhythmicity of cellular functions to support the circadian outputs in these regions and potentially drive nocturnality and diurnality. At the organism level, metabolism and foraging/feeding behavior have a reciprocal relationship with the clock. Hence, this has led to the hypothesis that energy balance can directly or indirectly influence the clock and may be one of several factors underlying temporal niche switches.^4–8^ The circadian thermoenergetics (CTE) hypothesis supports the idea that nocturnal endothermic animals shift their activity patterns from night to day when facing energetic challenges.^5^ This adaptation occurs because being active during the day, when ambient temperatures are typically higher, is associated with reduced daily energy expenditure in small mammals under natural temperate conditions.^4–6,9^ A nocturnal-diurnal switch also requires the direct interaction between light and activity to switch. Thus, the masking response of light may be reshaped depending on energetic status to drive the light-activity relationship. In nature, many animals have the ability to adjust their temporal patterns of activity/behavior depending on environmental cues,^5,10–12^ including migratory species that anticipate seasonal changes in energy availability and migrate to new geographic locations^13^ as well as voles that anticipate winter arrival^11,14,15^ and house mice that adapt their activity patterns in response to population densities, food resources, and predation risk.^16,17^

To understand what biological principles drive the daily timing of activity, we used the “work-for-food” model.^6,8,18,19^ This experimental approach allowed us to manipulate energy balance and switch mice to be nocturnal or diurnal. By adjusting the amount of wheel-running activity required to receive a food pellet reward, we simulated varying levels of natural food scarcity. By using this procedure, the animals were allowed to “forage,” obtain food, and eat at all times of the day. This enabled us to mimic different levels of food availability, allowing us to study the impact of energy balance on temporal activity patterns. Moreover, it has previously been shown that calorie-restricted mice display increased daytime activity.^20–23^

Unlike the melatonin-deficient C57BL/6J mice, CBA/CaJ mice are melatonin proficient. Moreover, there is no diurnal-nocturnal transcriptome atlas of the brain outside the SCN. Therefore, we assessed the rhythmic changes in brain areas that receive direct or indirect input from the SCN and play crucial roles in regulating sleep-arousal, feeding-fasting, energy metabolism, thermoregulation, reward, and locomotor activity.^2,3^ To this end, we conducted a comprehensive analysis of the rhythmic transcriptome in 21 tissues, including 17 brain regions, from nocturnal and diurnal male CBA/CaJ mice (492 samples passed quality control). Among these brain regions, seven (arcuate nucleus [ARC], brainstem [BRS], cerebellum [CER], cortex [COR], dorsomedial hypothalamus [DMH], hippocampus [HIP], and SCN) have been previously examined in time-series bulk RNA-sequencing (RNA-seq) studies in mice.^24–27^ We specifically chose to use male mice for our study, as females are more resilient in maintaining a nocturnal phenotype when faced with energetic challenges.^17,18^ Our dataset allowed us to compare the phases of rhythmic components in 14 overlapping tissues between nocturnal/diurnal mice and diurnal baboons (*Papio anubis*), which diverged from humans approximately 24 million years ago.^28^ Our analysis revealed distinct gene expression signatures, including clock genes, in various hypothalamic, thalamic, and cortical structures of nocturnal and diurnal mice. The rhythmic gene expression across tissues displayed different sets of genes with minimal overlap between the two groups. Diurnal mice exhibited shifted phases and reduced amplitudes of rhythmic gene expression. Overall, these findings offer valuable insights into the complex molecular dynamics underlying diurnal and nocturnal phenotypes and shed light on the differential gene expression patterns in key brain regions involved in regulating various physiological processes.

## RESULTS

### Switching mice to be nocturnal or diurnal by working for food

**Figure 1.**
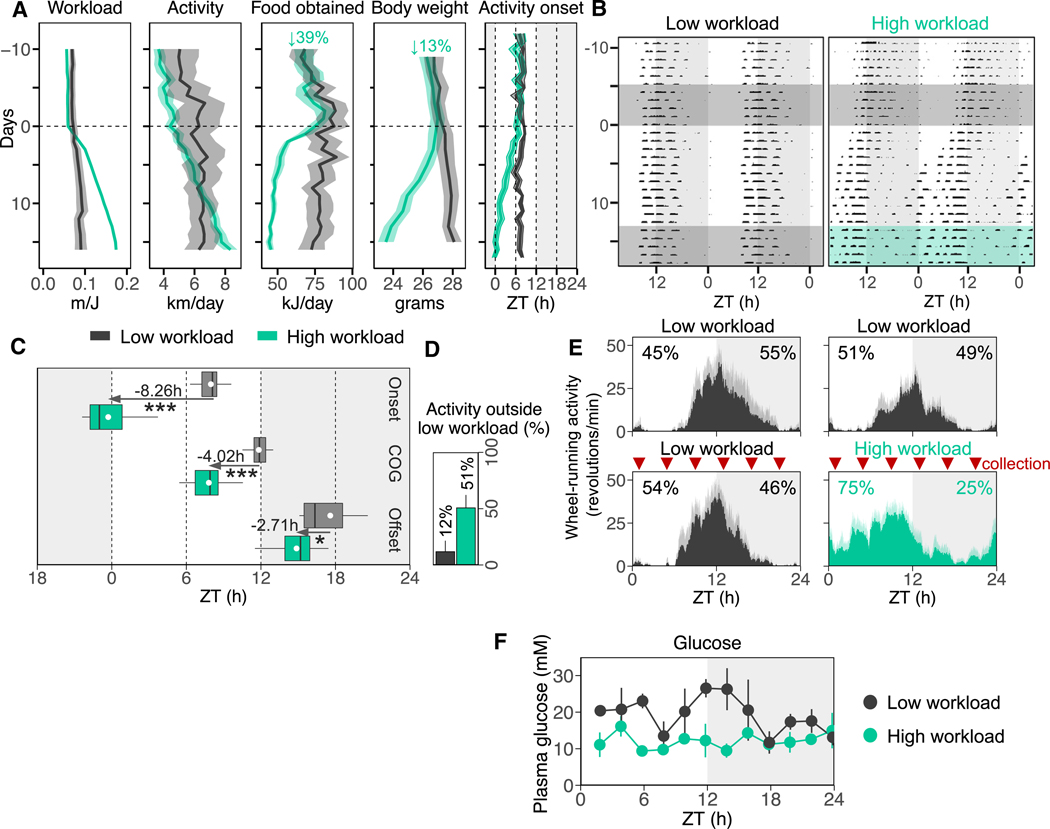
Switching mice to be nocturnal or diurnal by working for food (A) Gradual changes in workload (m/J), wheel-running activity, food obtained, body weight, and activity onset in low-workload (LWL) mice (gray) and high-workload (HWL) mice (green). The shaded areas indicate the dark phase (zeitgeber time [ZT] 12–ZT24). Data are represented as the mean ± SEM. (B) Representative double-plotted actograms of an LWL mouse (left) and an HWL mouse (right). Dark-gray shaded areas illustrate LWL condition, green shaded area illustrates HWL condition. (C) Onset, center of gravity (COG), and offset of wheel-running activity are shown for LWL and HWL animals. Phase shifts (advances) are depicted in hours. Mean values for each group are shown as white circles. *p < 0.05, **p < 0.01, ***p < 0.001. (D) Percentage activity that falls outside of the average active period of LWL mice. (E) Average daily wheel-running activity profiles for LWL (gray) and HWL (green) animals 5 days prior to the start of the protocol and the last 5 days of the protocol before tissue collection. Data are represented as the mean ± SD. Percentage diurnal and percentage nocturnal activity are shown. Red arrows indicate the timing of tissue collection (ZT1, 5, 9, 13, 17, and 21). (F) Plasma glucose levels in HWL and LWL animals are shown for 12 time points across the 24-h LD cycle. Data are represented as the mean ± SEM. See also Figure S1.

To induce daytime activity in a nocturnal mouse strain, we introduced a gradual food scarcity in the laboratory by coupling wheel-running activity with food availability, known as the work-for-food paradigm.^6,8,18^ After 10 days of habituation, during which mice were rewarded a 45-mg food pellet per 100 revolutions, the equivalent to 6.8 m/kJ, the mice were randomly assigned into two groups. In the high-workload (HWL) group, the workload was increased daily by 20 revolutions per food pellet (1.4 m/kJ) for the first 3 days. Subsequently, the workload was increased by 10 revolutions per pellet (0.7 m/kJ) each day, gradually inducing a negative energy balance (Figure 1A). The low-workload (LWL) group is similar to the *ad libitum* condition because food pellets were always present inside the cage. These LWL mice maintained their temporal activity pattern. Despite comparable total activity levels between the two groups, the HWL mice exhibited a progressively earlier onset of activity, progressively advancing into the light phase. By day 10, the HWL mice were primarily active during the day, and this pattern continued in the following days (Figures 1A and 1B). By day 16, the HWL mice showed a trend toward higher daily activity levels compared with LWL mice (Figure 1A). The HWL mice experienced a 39% reduction in daily food obtained (day 0 75 ± 5 kJ/day [mean ± SEM] vs. day 16 46 ± 2 kJ/day, n = 24, p < 0.001), a 13% decrease in body weight (day 1 27.0 ± 0.5 g vs. day 15 23.5 ± 0.5 g, n = 24, p < 0.001), and a 24% increase in daytime activity (Figures 1A and 1E).

This nocturnal-diurnal switch originated from an ~8-h phase advance in activity onset (LWL 7.97 ± 0.33 h vs. HWL 23.71 ± 0.58 h, n = 22, p < 0.001; Figures 1C and S1A), an ~4-h phase advance of the center of gravity (COG) (LWL 11.83 ± 0.28 h vs. HWL 7.81 ± 0.45 h, n = 22, p < 0.0001; Figures 1C and S1B), and an ~3-h phase advance in activity offset (LWL 17.58 ± 0.97 h vs. HWL 14.87 ± 0.50 h, n = 22, p < 0.02; Figures 1C and S1C). These changes resulted not only in a shift of the activity phase but also in a 5.5-h extension of the active period (Figure 1C), which is consistent with previous studies.^6^ Although LWL mice distributed 50% of their activity during the light phase and 50% during the dark phase, toward the end of the paradigm, 75% of the activity in HWL mice occurred during the light and only 25% during the dark phase. Fifty-one percent of the activity in HWL mice occurred outside the active period of LWL mice (Figure 1D). Therefore, LWL mice were classified as nocturnal and HWL mice as diurnal. As a result of the HWL protocol, plasma glucose levels were ~40% lower throughout the 24-h light-dark (LD) cycle (Figure 1F). This experimental paradigm reproduced what has been observed before^6,8,18^ and offers a laboratory model of temporal niche switching within a species.

### The rhythmic SCN transcriptome in nocturnal (LWL) and diurnal (HWL) mice

**Figure 2.**
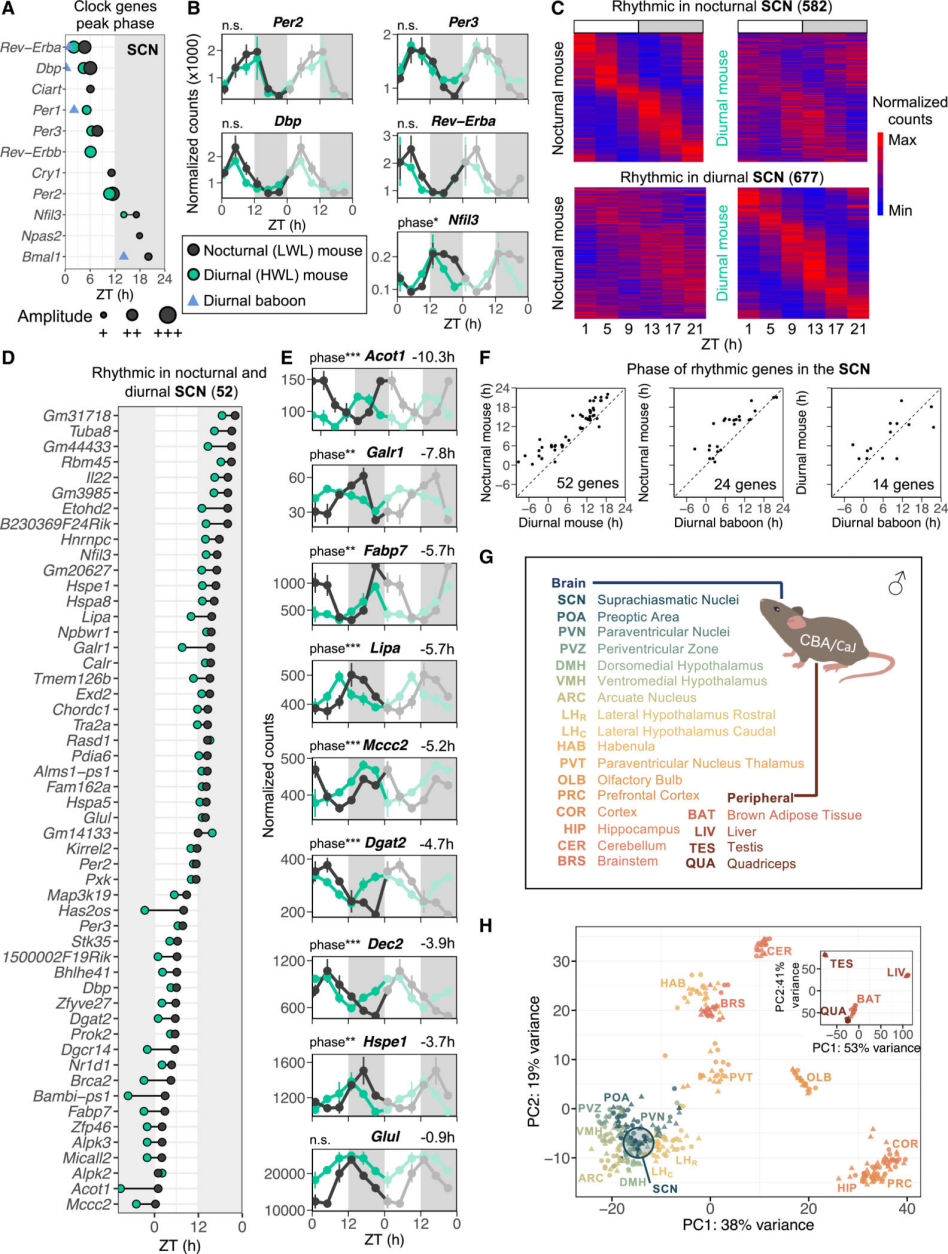
The rhythmic SCN transcriptome in nocturnal (LWL) and diurnal (HWL) mice (A) Peak phase of rhythmic clock genes in the SCN of nocturnal mice (black), diurnal mice (green), and diurnal baboons (blue triangles). Data are shown only for statistically significant rhythmic clock genes. Amplitude is defined by point size (for mice only). The shaded area indicates the dark phase (ZT12–ZT24). (B) Double-plotted expression profiles for a selection of clock genes. Data are represented as the mean ± SEM. (C) Heatmap of normalized gene expression of rhythmic genes in the SCN of nocturnal and diurnal mice (false discovery rate [FDR] < 0.05) across six time points. Max and Min represent the relative ranked maximum and minimum values for the specific gene, respectively. (D) Phase of peak expression of common rhythmic genes in nocturnal and diurnal mice in the SCN. (E) Double-plotted expression patterns for a selection of genes that show major phase advances in diurnal mice. Phase shifts in hours are shown on top of each plot and labeled for significant differences in phase, with *p < 0.05, **p < 0.01, ***p < 0.001. (F) Correlations between the phase of rhythmic gene expression in the SCN of nocturnal mice, diurnal mice, and diurnal baboons. (G) List of tissues collected (17 brain sites, 4 peripheral organs). (H) Principal-component analysis (PCA) was performed on all six time points of nocturnal and diurnal mice for brain and peripheral tissues separately. Circles represent nocturnal mice; triangles represent diurnal mice. See also Figures S1–S4 and Tables S1, S2, and S3.

To comprehensively analyze the transcriptional state of the SCN in nocturnal (LWL) and diurnal (HWL) mice, we conducted unbiased RNA-seq to capture transcripts with approximately 24-h rhythms. Tissue samples were collected from nocturnal (LWL) and diurnal (HWL) mice at 4-h intervals over a 24-h period, denoted as zeitgeber time (ZT) 1, 5, 9, 13, 17, and 21, with ZT0 being when the lights turn on and ZT12 when the lights turn off. Transcripts of several clock genes passed the statistical threshold of cycling in the diurnal and/or nocturnal mice; mRNAs of *Per2*, *Per3*, *Dbp*, *Rev-Erba*, and *Nfil3* were detected as rhythmic under both conditions (Figures 2A and 2B). The phase of peak expression of these transcripts advanced in the diurnal (HWL) mice. However, the magnitude of these advances (*Per2* and *Per3* 1 h, *Dbp* 2 h, *Rev-Erba* and *Nfil3* 3 h) relative to that in nocturnal (LWL) mice were smaller than the ~8-h advance in activity onset observed in diurnal (HWL) mice. This is in line with the expression of clock genes/proteins in the SCN of both nocturnal and diurnal species and niche-switched animals, which generally peak at a similar phase.^1,6,28–36^ Furthermore, in the SCN of the diurnal primate baboon (*Papio anubis*), the phases of these clock genes were similar to those in both diurnal (HWL) and nocturnal (LWL) mice, with the exception of *Bmal1*, which peaked 6 h earlier (Figures 2A and S2A).^28^ The stable expression patterns of clock genes in the SCN suggest that the circadian molecular SCN clock is relatively resilient to temporal niche switching within the species.

In an unbiased assessment of the SCN rhythmic transcriptome, we identified 582 rhythmic transcripts in nocturnal (LWL) mice and 677 rhythmic transcripts in diurnal (HWL) mice (Figure 2C; Tables S1 and S2). Only 52 transcripts (<10%), including the above-described five clock genes, were rhythmic under both conditions (Figure 2D). This indicates that the nocturnal and diurnal phenotypes are associated with distinct sets of rhythmic genes in the SCN. Functional annotation of the rhythmic transcripts revealed distinct biological processes that were temporally organized in the SCN of nocturnal (LWL) and diurnal (HWL) mice. The top six Gene Ontology (GO) terms enriched in the nocturnal (LWL) mice were protein folding, circadian rhythm, brain development, transcription, tissue homeostasis, and lipid homeostasis, and in the diurnal (HWL) mice, they were circadian rhythm, RNA splicing, catabolic process, cell-cycle regulation, tRNA modification, and insulin receptor signaling (Table S3).

Although the phases of mRNA rhythms in clock genes that cycled under both conditions were similar between nocturnal (LWL) and diurnal (HWL) mice, 44 of 47 other common rhythmic genes in the SCN of diurnal (HWL) mice phase advanced, with an average phase advance of 4.2 ± 0.4 h (Figure 2D). This phase shift was similar to the shift in wheel-running activity (COG −4.0 h; Figure 1C) and yet larger than the phase shifts of the core clock components (−1.8 ± 0.4 h; Figures 1A and S2C) that remained rhythmic under both conditions. To assess whether those common rhythmic genes are controlled by the circadian clock, we compared those genes with another SCN transcriptome dataset from wild-type mice held under constant darkness (DD).^37^ From this comparison we found that 38 of those 47 common rhythmic genes in the SCN were not rhythmic in *ad libitum*-fed mouse SCN of dark controls from another study.^37^ Furthermore, *Cry1*^−/−^*;Cry2*^−/−^ mice synchronized to a light-dark cycle on a high workload also shift from nocturnal to diurnal.^18^ These findings imply that these non-clock-component common cyclers likely enabled the SCN to adapt to the temporal niche switching. Among the rhythmic transcripts that phase advanced in the SCN, several encoded proteins involved in lipid and amino acid metabolism (Figures 2D and 2E): fatty acid (FA) uptake and transport (brain fatty acid binding protein [*Fabp7*]), formation of triglycerides from diacylglycerol and fatty acyl-CoA (*Dgat2*), conversion of acyl-CoAs to fatty acids and CoA (*Acot1*), lysosomal hydrolysis of cholesteryl esters and triglycerides (*Lipa*), and breakdown of branched-chain amino acid leucine to eventually yield acetyl CoA and acetoacetate (3-methylcrotonoyl-CoA carboxylase; *Mccc2*). The opposite phasing of *Lipa* and *Dgat2* and the advanced phase of these enzyme-coding transcripts indicate a shift in triglyceride breakdown and synthesis in the SCN of diurnal (HWL) mice. The galanin receptor (*Galr1*), implicated in mediating the glucoregulatory function of the galanin class of neuropeptides,^38^ was also rhythmic and phase advanced in diurnal (HWL) mice. Glutamate-ammonia ligase (*Glul*), responsible for detoxifying excess glutamate and ammonia in the brain,^39^ was also phase advanced in the SCN. Interestingly, heat-shock proteins (*Hspe1*, *Hspa8*, and *Hspa5*) were also phase advanced in the SCN. Although prior studies have indicated the resistance of the SCN clock to temperature fluctuations,^40,41^ our findings suggest that the heat-shock pathway in the SCN aligns with the reduction in core body temperature in HWL diurnal mice.^8^ This implies that the changes in body temperature resulting from energy deficiency might have contributed to the observed changes in gene expression within the SCN. Finally, *Dec2* (*Bhlhe41*), a basic regulator of the mammalian molecular clock and sleep,^42^ is phase advanced with 3.9 h in diurnal (HWL) mice.

Comparing the rhythmic genes in the SCN of diurnal baboons to nocturnal (LWL) mice, we observed that the phase of 21 of 24 rhythmic genes (88%) peaked earlier in baboon SCN (Figure 2F), although compared with diurnal (HWL) mice, we observed that certain genes in the baboon SCN exhibited earlier peaks, while others displayed later peaks compared with diurnal (HWL) mouse SCN. In summary, while the core clock genes in the SCN were relatively resilient to temporal niche switching, the temporal niche-specific rhythmic transcripts and the phases of common rhythmic transcripts revealed in this study offer insights into how the SCN may adapt or respond to niche switching and energy deficiency.

### Clock-gene expression changes across tissues in nocturnal (LWL) and diurnal (HWL) mice

To examine the tissue-specific molecular signature associated with the nocturnal-diurnal switch, we assessed diurnal gene expression profiles with mRNA-seq of 492 samples across 21 different tissues, including 17 brain regions and 4 peripheral tissues, in nocturnal (LWL) and diurnal (HWL) mice (Figures 2G and S1D–S1G). Tissue samples were collected at 4-h intervals over a 24-h period, denoted as ZT1, 5, 9, 13, 17, and 21, with ZT0 being when the lights turn on and ZT12 being when the light turns off. We detected 12,330–20,463 transcripts per tissue (Tables S4 and S5). Principal-component analysis (PCA) based on gene expression levels showed clustering patterns of: (1) hypothalamic regions centered around the SCN, (2) habenula (HAB) and BRS, (3) HIP and cortical structures, and (4) peripheral tissues (Figures 2H and S3). Further PCA of individual tissues showed separation by phenotype for most tissues (Figure S4). Overall, these analyses showed that the nocturnal and diurnal phenotypes were linked with specific gene expression profiles.

**Figure 3.**
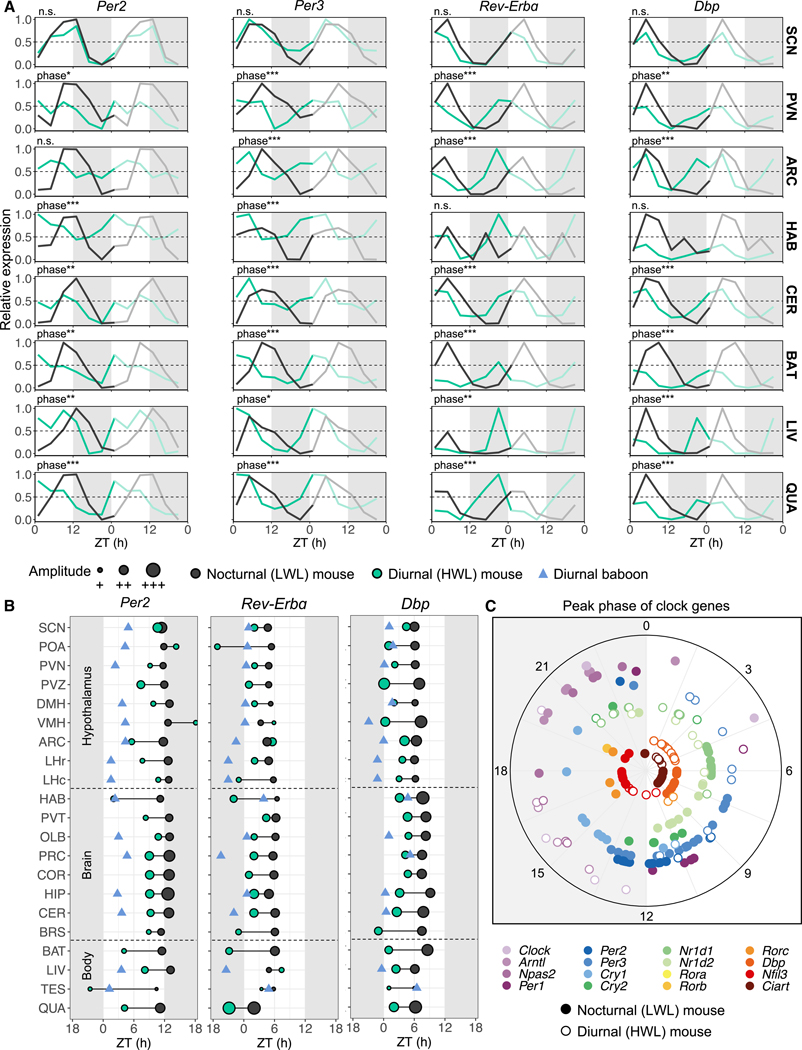
Clock-gene expression changes across tissues in diurnal (HWL) mice (A) Double-plotted relative expression profiles of *Per2*, *Per3*, *Rev-Erbɑ*, and *Dbp* in nocturnal mice (black) and diurnal mice (green), across a selection of five brain sites (suprachiasmatic nucleus, SCN; paraventricular nucleus, PVN; arcuate nucleus, ARC; habenula, HAB; and cerebellum, CER) and three peripheral organs (brown adipose tissue, BAT; liver, LIV; and quadriceps, QUA). Data are represented as the mean. Phase shifts are labeled for significant differences in phase, with *p < 0.05, **p < 0.01, ***p < 0.001. The shaded areas indicate the dark phase (ZT12–ZT24). (B) Phases of peak expression of a selection of clock genes, *Per2*, *Rev-Erbɑ*, and *Dbp*, in nocturnal mice (black), diurnal mice (green), and diurnal baboons (blue triangles) across all tissues. Data are shown only for statistically significant rhythmic clock genes. Amplitude is displayed by point size. (C) Radial plot showing the peak phases of significantly rhythmic clock genes across tissues. See also Figure S2 and Tables S1 and S6.

To test if, in contrast to the SCN, the phase of extra-SCN brain sites and peripheral organs is altered in niche-switched mice, we analyzed clock-gene expression patterns across all 21 tissues collected from both nocturnal (LWL) and diurnal (HWL) mice. Transcripts of several clock genes and their immediate outputs were detected as rhythmic in the diurnal and/or nocturnal mice: mRNAs of *Bmal1*, *Per2*, *Per3*, *Rev-Erbα*, *Rev-Erbβ*, *Dbp*, *Nfil3*, and *Ciart* passed the statistical threshold of rhythmic in at least 14 tissues. Interestingly, we found fewer rhythmic clock genes in diurnal (HWL) mice (Figure S2C). In diurnal (HWL) mice, the peak expression of rhythmic clock genes was phase advanced compared with nocturnal (LWL) mice (Figures 3A–3C and S2C; Table S6). The magnitude of these phase shifts was on average larger in extra-SCN brain regions (4.1 ± 0.3 h) compared with the SCN (1.8 ± 0.4 h), but smaller than in peripheral tissues (5.8 ± 0.5 h) (Figure S2C). These findings suggest that the molecular clocks in the extra-SCN regions adapt to temporal niche switching. For example, in diurnal mice, *Per2*, *Rev-Erbα*, and *Dbp* expression was phase advanced with several hours in most non-SCN tissues (Figures 3A–3C). The peak phases of clock genes were more stable across tissues in nocturnal (LWL) mice, whereas diurnal (HWL) mice showed greater variability in peak phases across tissues (Figures 3B and 3C). This implies that the molecular clocks of different brain sites and peripheral organs respond with varying degrees to temporal niche switching, highlighting tissue-specific adaptations.

We next compared the clock-gene expression profiles with that of diurnal baboons and observed a greater overlap between diurnal (HWL) mice and baboons compared with nocturnal (LWL) mice and baboons (Figure 3B). In summary, the rhythmic expression of clock genes phase advanced or became arrhythmic in diurnal (HWL) mice. The loss of clock-gene rhythmicity may be the result of a dampened amplitude of the rhythm, which might be caused by increased variation between animals or desynchronization of cells within a tissue. It is possible that different cell types within the tissue respond differently to the diurnal phenotype, which may lead to a weakened circadian signal with a reduced amplitude observed in the gene expression patterns of diurnal (HWL) mice.

### Rhythmic gene expression changes across tissues in nocturnal (LWL) and diurnal (HWL) mice

**Figure 4.**
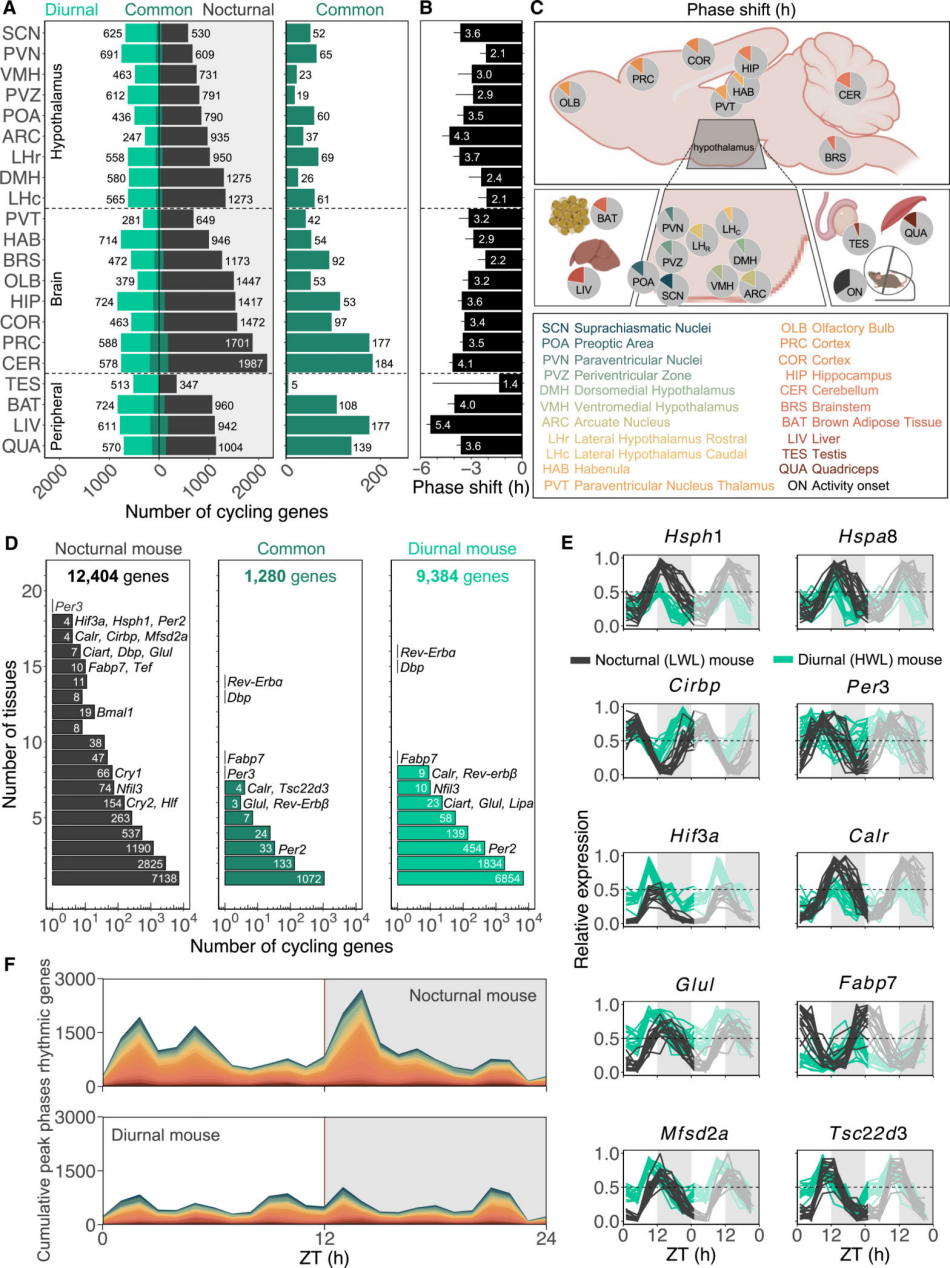
Rhythmic gene expression changes across tissues in nocturnal (LWL) and diurnal (HWL) mice (A) The bar graph indicates the number of genes rhythmic only in diurnal mice (light green), common rhythmic genes (dark green), and genes rhythmic only in nocturnal mice (gray) for each tissue. (B) The average phase shift ± SEM in hours of all common rhythmic genes per tissue. (C) Pie charts (h/24 h) represent the average phase shift of all common rhythmic genes for each tissue at the anatomical location. Created with http://biorender.com. (D) Distribution of rhythmic genes and the number of tissues in which they are rhythmic in nocturnal mice, in common, and in diurnal mice. (E) Double-plotted normalized expression profiles for a selection of genes across 17 brain sites in nocturnal mice (gray) and diurnal mice (green). (F) Cumulative peak phases of expression of rhythmic genes in all different tissues of nocturnal mice and diurnal mice. The shaded areas indicate the dark phase (ZT12–ZT24). See also Figures S5 and S6; Tables S2, S3, S7, and S14.

Metabolism is known to interact with the circadian clock to influence the amplitude and phase of rhythmic gene expression.^43^ To further assess the broader impact of a diurnal-nocturnal phenotype on the circadian gene network, we next examined the whole rhythmic transcriptome of the collected tissues. Across all tissues, a subset of genes displayed rhythmic expression, ranging from 284 to 2,171 depending on the tissue and condition (Figures 4A and S5; Table S2). Diurnality was associated with a reduction in the overall rhythmicity of gene expression in the majority of tissues (Figures 4A and S5). In general, there were more rhythmic genes detected in brain areas other than the hypothalamus. Most rhythmic genes were found in the CER (2,749), while the least rhythmic genes were found in the testis (865) (Figures 4A and S5). Only a small subset of genes (5–184 genes per tissue) was commonly cycling in both phenotypes (Figure 4A), indicating that distinct gene sets were associated with the nocturnal and diurnal phenotypes. To further examine whether the phase of gene expression was shifted in diurnal (HWL) animals, we assessed the peak phases of common cycling genes. Across tissues, the majority of these genes phase advanced in diurnal (HWL) mice, with average phase shifts ranging from 1.4 h in testis to 5.4 h in the liver (LIV) (Figures 4B and 4C).

A total of 12,404 rhythmic transcripts were detected in nocturnal (LWL) mice compared with 9,384 rhythmic transcripts in diurnal (HWL) mice, of which 1,280 were detected as rhythmic in both phenotypes (Figure 4D; Table S7). Functional annotation of rhythmic transcripts in five or more tissues revealed distinct pathways associated with each phenotype. In nocturnal (LWL) mice, pathways related to protein folding (*Hsph1*, calreticulin [*Calr*], and *Hspa8*), circadian rhythm (*Per3*, *Ciart*, *Tef*, and *Hlf*), and mRNA processing (*Cirbp*) displayed rhythmic expression (Table S3), consistent with previous transcriptomic data from nocturnal mice.^24^ On the other hand, diurnal mice (HWL) exhibited rhythmicity in pathways involved in circadian rhythm, mRNA metabolic process, and catabolic process (Table S3). Several of those rhythmic genes show remarkably similar/synchronized cycling expression patterns throughout the brain, and these patterns changed in diurnal (HWL) mice (Figure 4E). For example, the temperature-sensitive heat-shock proteins (*Hsph1* and *Hspa8*) and the cold-inducible RNA-binding protein (*Cirbp*) phase advanced in their expression patterns and there-fore align with the reversed core body temperature patterns observed in diurnal (HWL) mice.^8^ *Per3*, the most universally rhythmic gene among tissues (rhythmic in 19 of 21 tissues), also phase advanced. In contrast, *Per2* lost its rhythmicity in the majority of tissues in diurnal (HWL) mice, while *Rev-Erba* and *Dbp* remained cycling in most tissues under both conditions (Figure 4D). Hypoxia-inducible factor 3 subunit a (*Hif3a*), a component of a transcription factor that regulates adaptive responses to low oxygen and is implicated in sleep deprivation,^26^ was upregulated and phase advanced in diurnal (HWL) mice. The lengthening of the active period in diurnal (HWL) mice indicates that they were indeed sleep deprived. *Calr* encodes a protein involved in regulating cellular calcium levels^44^ and was downregulated during nighttime in diurnal (HWL) mice. *Glul* encodes an enzyme responsible for synthesizing glutamine from glutamate and was upregulated and phase advanced in diurnal (HWL) mice, implying altered metabolic regulation of glutamine throughout the brain in response to energy deprivation. *Fabp7*, which is implicated in fatty acid uptake in astrocytes and appeared to be also involved in human/mouse/fly sleep,^45,46^ was phase advanced with ~6 h with reduced amplitude in diurnal (HWL) mice. *Mfsd2a*, a transport protein responsible for omega-3 fatty acid uptake through the blood-brain barrier,^47^ phase advanced and upregulated during the light phase in diurnal (HWL) mice. Finally, *Tsc22d3*, encoding an immunosuppressive protein stimulated by glucocorticoids, was upregulated at the beginning of the light phase, showing a potential link between energy deficiency and immune function.

To determine the phase of rhythmic gene expression in each tissue of both nocturnal (LWL) and diurnal (HWL) mice, we next analyzed the temporal distribution of peak phases of cycling genes in each condition. The distribution of rhythmic transcripts changed in diurnal (HWL) mice with a distinct pattern specific to each tissue (Figure S6). Some tissues shifted in phase (periventricular zone [PVZ] and habenula), while others were reduced in amplitude (DMH, ARC, bulbus olfactory [OLB], and CER). Rhythmic gene expression across tissues in nocturnal (LWL) mice was synchronized, revealing three prominent peaks (ZT2, 5, 14) and three minor peaks (ZT10, 17, and 21) (Figure 4F). However, such distinct peaks were not observed in diurnal (HWL) mice. Instead, similar phased peaks were reduced in amplitude by 60%. Taken together, these universal changes in rhythmic gene expression indicate a tissue-wide dampening of rhythmic transcripts with an ~24-h period as a response to a niche switch.

### The transcriptome of feeding-fasting and sleep-wake centers in nocturnal (LWL) and diurnal (HWL) mice

**Figure 5.**
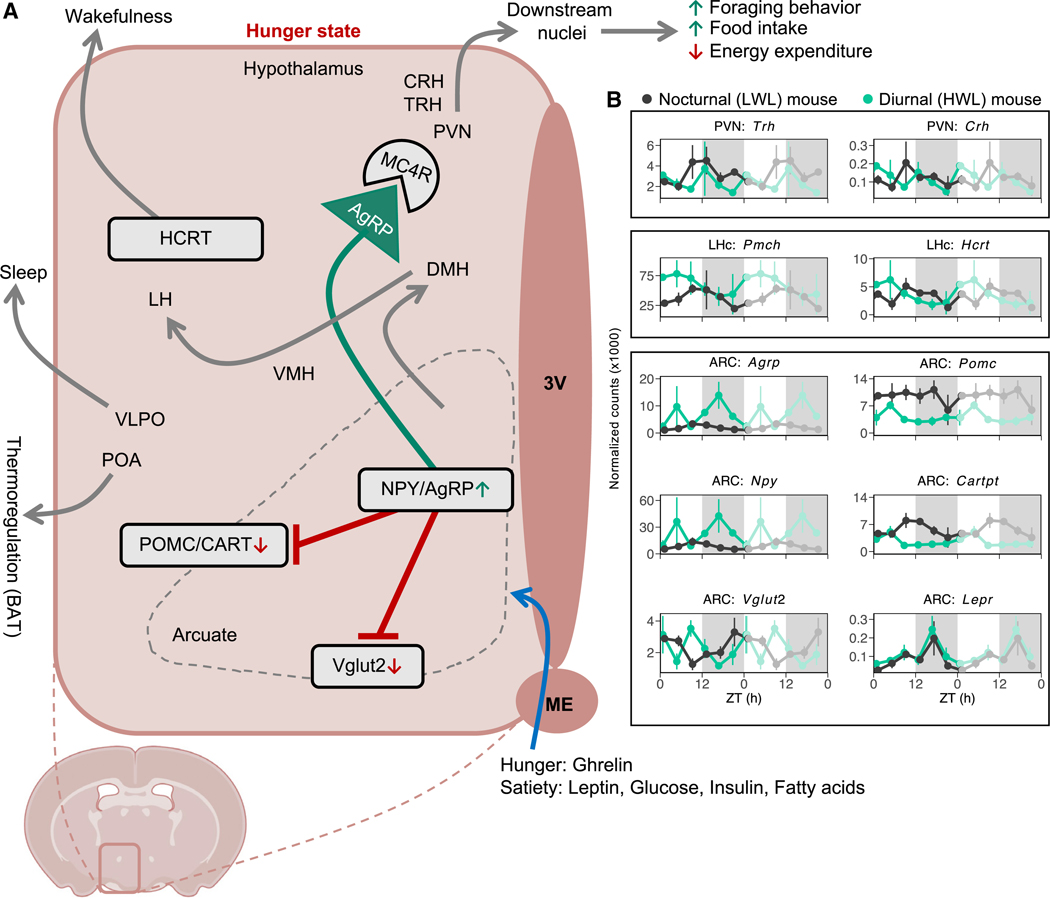
The transcriptome of feeding-fasting and sleep-wake centers in niche-switched mice (A) Schematic representation of hypothalamic hunger-satiety and sleep-wake centers and neuropeptides involved in control of feeding behavior and energy expenditure. 3V, third ventricle; ME, median eminence. (B) Double-plotted normalized expression profiles for candidate genes in the PVN, LHc, and ARC of nocturnal mice (black) and diurnal mice (green). Data are represented as the mean ± SEM. The shaded areas indicate the dark phase (ZT12–ZT24).

To investigate the impact of negative energy balance on hungersatiety and sleep-wake circuits, we conducted a focused analysis of candidate genes within specific hypothalamic centers (Figure 5A). As expected, we observed a significant upregulation of orexigenic (appetite-promoting) genes, including arcuate neuropeptide y (*Npy*) and agouti-related protein (*Agrp*), in energetically challenged mice (Figure 5B). These genes showed remarkably similar expression patterns, suggesting their origin in the same neuronal population. In contrast, the expression of anorexigenic (appetite-suppressing) genes, such as proopiomelanocortin (*Pomc*) and Cart prepropeptide (*Cartpt*), was downregulated in energetically challenged mice. Vesicular glutamate transporter 2 (*Vglut2*), which is responsible for glutamate uptake into synaptic vesicles of excitatory neurons, was downregulated during peak expression of NPY/AgRP. In addition, we observed an upregulated and phase-advanced rhythm of pro-melanin-concentrating hormone (*Pmch*) in the lateral hypothalamus (LHc), which promotes eating behavior during day-time in energy-depleted mice. Neuropeptides originating from the ARC play a crucial role in conveying information about energetic status. These neuropeptides bind to melanocortin 4 receptor (MC4R) expressed in the paraventricular nucleus (PVN), which acts on downstream nuclei to modify behavior, including increasing foraging and food intake, as well as reducing energy expenditure when resources are limited. The hypothalamic neuropeptide hypocretin/orexin (*Hcrt*) is a major regulator of sleep and arousal.^48,49^ In diurnal (HWL) mice, we observed a phase advance of *Hcrt*, aligning with wakefulness. When food availability is limited, animals tend to extend their active period to increase the likelihood of encountering food, which is crucial for survival.^50,51^ Interestingly, mice lacking orexin neurons do not show this behavioral adaptation,^50^ suggesting that orexin neurons may be a component of the nocturnal-diurnal switch by interacting with feeding centers when energy reserves are low. By utilizing these hypothalamic pathways during food scarcity, animals can adjust their physiology and behavior to conserve energy and enhance survival.

### Negative energy balance leads to common and tissue-specific changes in gene expression

**Figure 6.**
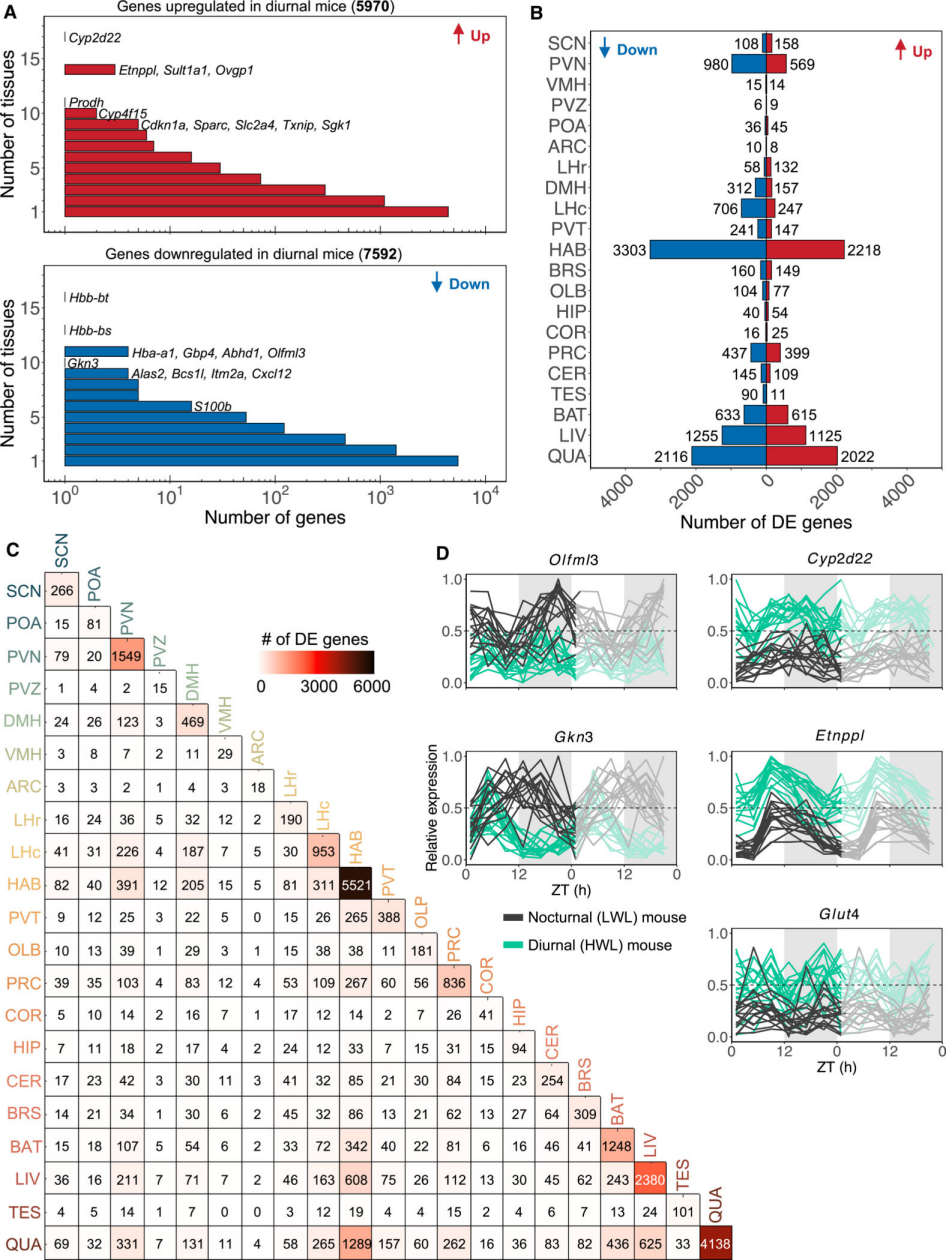
Negative energy balance leads to systemic and tissue-specific changes in gene expression (A) Distribution of differentially expressed (DE) genes and the number of tissues in which they are DE. (B) Number of DE genes for all tissues. Downregulated genes are depicted in blue, and upregulated genes are in red. (C) Tissue-by-tissue overlap of DE genes in each tissue. (D) Double-plotted normalized expression profiles of a selection of DE genes across 17 brain sites in nocturnal mice (gray) and diurnal mice (green). The shaded areas indicate the dark phase (ZT12–ZT24). See also Figure S7; Tables S8, S9, S10, and S11.

To assess the overall impact of negative energy balance on gene expression across different tissues, we performed a differential analysis comparing nocturnal (LWL) and diurnal (HWL) mice, independent of time of day. A total of 12,063 unique differentially expressed (DE) genes were detected across all tissues (Tables S8, S9, and S10). Among these genes, 5,970 were upregulated and 7,592 were downregulated in one or more tissues. Seventy-one genes were consistently upregulated, and 90 genes were consistently downregulated in five or more tissues of energy-deficient mice (Table S10). Interestingly, only one DE gene (*Cyp2d22*) was shared among all brain sites. Several genes DE in nine or more tissues are involved in processes such as glucose transport (*Glut4* [*Slc2a4*]), lipid metabolism (*Etnppl*), xenobiotic/metabolite degradation (*Cyp2d22* and *Cyp4f15*), mitochondrial function (*Prodh* and *Bcs1l*), apoptosis (*Cdkn1a*), and the cellular stress response (*Sgk1*) (Figure 6A; Table S13). For instance, the glucose transporter gene *Glut4* (*Slc2a4*) was consistently upregulated across nine brain regions of energy-deficient mice (Figure 6D). This upregulation may reflect the increased energy demands for firing neurons in these specific brain structures. The sustained reduction in blood glucose levels induced by the energy deficiency in these mice (Figure 1F) could potentially lead to alterations in glucose transporter expression and function throughout the brain, ensuring an adequate energy supply to active neuronal circuits. In addition, other cerebral glucose transporters, such as *Glut1/2* (*Slc2a1/2*), were specifically upregulated in the habenula and PVN, respectively, in response to energy deficiency. It is known that *Glut4* is primarily expressed by neurons, while *Glut1* is predominantly expressed by astrocytes/tanycytes/ependymal cells to facilitate D-glucose uptake across the blood-brain barrier.^52^ Given that diurnal (HWL) mice immediately revert their activity to the previous nocturnal phase upon *ad libitum* food supplementation,^8^ accompanied by a rapid rise in glucose levels, we speculate that glucose, the primary energy source for neurons and glial cells, may serve as a viable candidate signaling molecule to the brain.

Furthermore, genes associated with oxygen transport (*Cyp2d22* and *Cyp4f15*) and hypoxia (*Sult1a1*, *Txnip*, *Sparc*, and *Hyou1*) were upregulated, indicating reduced oxygen delivery to the brains of energy-depleted mice and potential hypoxic conditions. Another observation was the elevated expression of the mitochondrial enzyme *Prodh* in energy-deficient mice. *Prodh* plays a crucial role in facilitating glutamate production through the breakdown of proline, a process that becomes activated in response to low nutrient availability, enabling cells to transition toward catabolic metabolism and acquire the necessary energy for cellular survival.^53^ *Etnppl*, a fasting-induced gene involved in lipid homeostasis in astrocytes, was induced in energy-deficient mice (Figure 6D). Notably, *Etnppl* is known to be stimulated by glucocorticoid,^54^ which is dramatically elevated in the plasma of mice at high workloads.^6^ Furthermore, *Olfml3*, a gene expressed in microglia and involved in the formation of extracellular matrix structure, was downregulated. *Gkn3*, a gene that is downregulated with sleep deprivation,^26^ was also downregulated in diurnal (HWL) mice that showed fragmented activity patterns. Last, multiple thalamic and cerebral brain sites (habenula, PRC, BRS, CER, COR, and OLB) exhibited reduced expression of astrocyte markers (*S100b* and *Aqp4*),^55^ indicating a potential loss of astrocytes.

Overall, these findings demonstrate that various regions of the brain exhibit partially similar responses to negative energy balance, as evidenced by global changes in gene expression. However, a closer examination of DE genes across tissues revealed distinct molecular responses specific to each tissue, with only a small subset of DE genes overlapping between them (Figures 6B and 6C; Table S11). There is considerable variation in the number of DE genes among tissues, with the PVZ, ARC, ventromedial hypothalamus (VMH), and COR being the least affected, while the habenula, quadriceps muscle (QUA), LIV, and PVN exhibit the most pronounced effects (Figures 6B, 6C, and S7). The PVN is known to play a critical role in integrating information regarding the energetic status and orchestrating downstream physiological responses.^56^ On the other hand, the exact involvement of the habenula in controlling energy homeostasis remains less clear. However, the high expression of several neuropeptides related to feeding in the habenula suggests its potential involvement in the regulation of feeding behavior and energy balance.^57^ The habenula, with 5,521 DE genes, was the most affected brain site, representing a 4–500 times higher number compared with other brain regions. Therefore, we further investigated the distinctive molecular signature of the habenula in nocturnal and diurnal mice.

### A potential role for the habenula in driving nocturnal-diurnal switches

**Figure 7.**
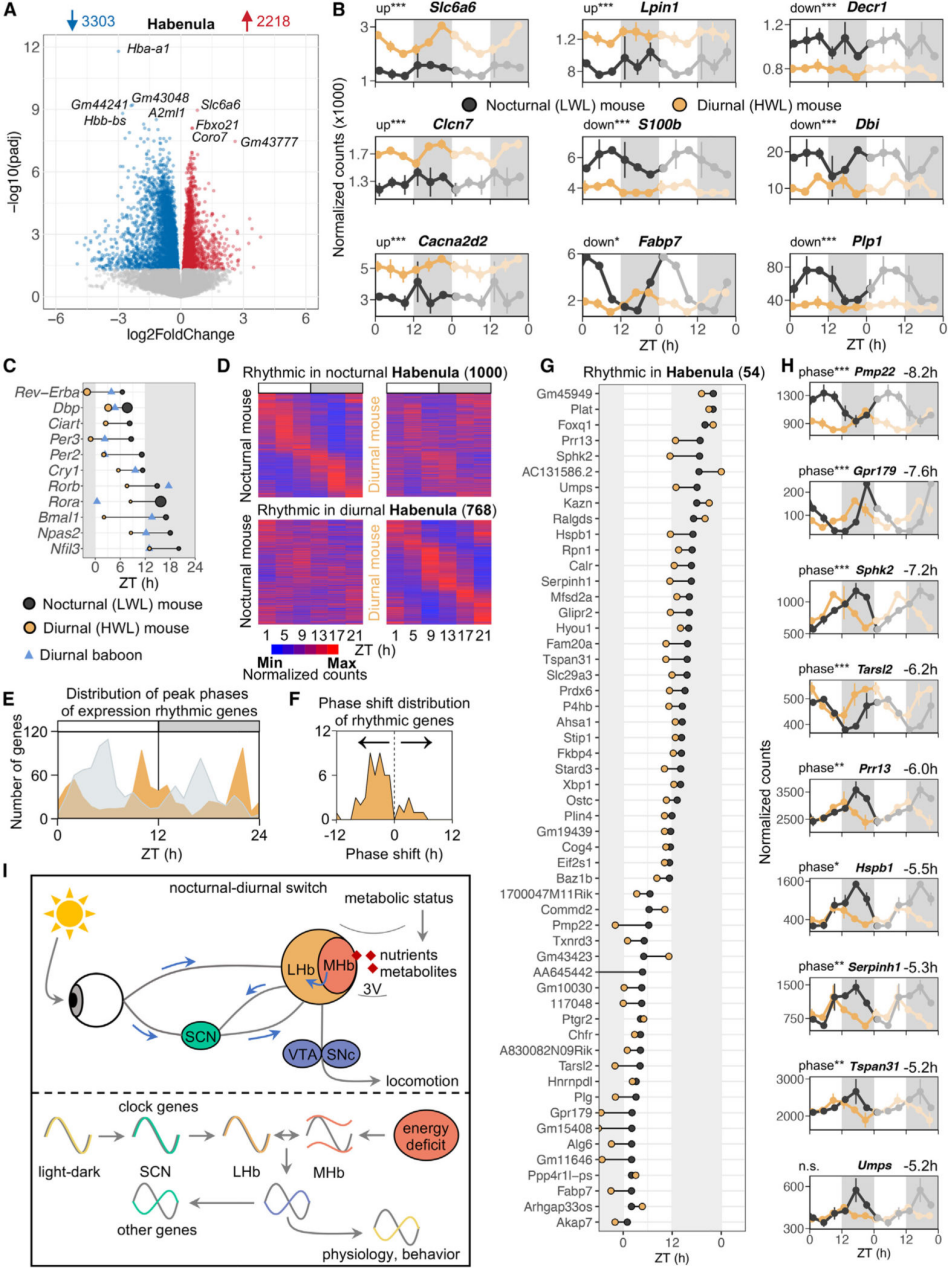
A potential role for the habenula in driving nocturnal-diurnal switches (A) Volcano plot of differentially expressed (DE) genes in the habenula. Upregulated genes in diurnal mice (red) and downregulated genes in diurnal mice (blue) are shown. (B) Double-plotted expression patterns for a selection of DE genes. Data are represented as the mean ± SEM. (C) Peak phase of clock genes in the habenula of nocturnal mice (black), diurnal mice (orange), and diurnal baboons (blue). Amplitude is defined by point size (for mice only). (D) Heatmaps of relative expression of rhythmic genes in the habenula of nocturnal and diurnal mice (FDR < 0.05) across six time points. Max and Min represent the relative ranked maximum and minimum values for the specific gene, respectively. (E) Temporal distribution of peak phases of expression of cycling genes in the habenula of nocturnal (gray) and diurnal (orange) mice. The y axis indicates the number of cycling genes that peak in expression at ZT1–ZT24 (1-h bins). (F) Phase shift distribution of genes that were rhythmic in both nocturnal and diurnal mice. (G) Phase of peak expression of common cycling genes in nocturnal (black) and diurnal (orange) mice. (H) Double-plotted expression patterns of a selection of genes that show major phase advances in diurnal mice. Phase shifts in hours are shown on top of each plot and labeled for significant differences in phase, with *p < 0.05, **p < 0.01, ***p < 0.001. The shaded areas indicate the dark phase (ZT12–ZT24). (I) Schematic of a hypothetical biological pathway; see discussion for more details. SCN, suprachiasmatic nucleus; LHb, lateral habenula; MHb, medial habenula; 3V, third ventricle; VTA, ventral tegmental area; SNc, substantia nigra pars compacta. See also Tables S3, S8, and S12.

Here, we discovered that the habenula is the brain region most responsive to a temporal niche switch, showing the highest number of DE genes (2,218 up and 3,303 down) and phase shifts in cycling genes (Figure 7). The habenula is a highly conserved vertebrate thalamic brain structure that occupies a central anatomical position and contains its own intrinsic circadian clock,^58–62^ which makes it an excellent candidate for driving the nocturnal-diurnal switch.

Functional annotation of DE genes revealed that upregulated genes in diurnal (HWL) mice were associated with histone modification, neuron projection, pluripotency, chromatin organization, and cell junction organization, while downregulated genes were associated with translation, oxidative phosphorylation, mitochondrial organization, ribonucleoprotein complex biogenesis, and cellular response to chemical stress (Table S3). A more targeted analysis revealed that DE genes in the habenula encoded neurotransmitter transporters (up: *Slc6a6*, *Slc6a1*, *Slc6a8*, and *Slc6a15*), ion channels (up: *Clcn6/7*, *Cacna2d2*, and *Kcnb1*), proteins involved in lipid metabolism (up: *Lpin1*; down: *Fabp3/5/7*, *Hacd1*, *Decr1*, *Dbi*, and *Elovl2/6*), and proteins involved in myelin metabolism (up: *Smpd3* and *Myrf*; down: *Trem2* and *Plp1*) (Figures 7A and 7B). Five hundred two genes encoding ion channels were either upregulated (354 genes) or downregulated (148 genes) in diurnal (HWL) mice (Table S12).

The ependymal cell layer of the medial habenula (MHb) forms a lining along the third ventricle of the brain, establishing direct contact with circulating cerebrospinal fluid (CSF). MHb tanycytes^63^ are likely capable of sensing nutrients and metabolites in the circulation, enabling communication with downstream habenular neurons. To investigate potential signaling molecules that could be recognized by the MHb, resulting in the observed transcriptomic changes, we focused on nutrient/metabolite receptors and transporters. Diurnal (HWL) mice exhibited altered expression patterns of several genes involved in fatty acid homeostasis (*Fabp3/5/7*, *Elovl2/6*, *Fto*, and *Fads2*), suggesting a potential shift in fatty acid utilization by the brain and aligning with the observation that HWL mice start to shift their activity toward diurnality when weight loss occurs (Figure 1). However, our current data cannot establish whether the shift in fatty acid metabolism is a cause or a consequence of the behavioral shift. Among the most significantly upregulated genes were taurine transporters (*Slc6a6*, *Lrrc8d*, and *Slc36a1*). Taurine uptake can be regulated by hypoxia, of which several markers were affected in the habenula (up: *Hif3a*, *Arnt2*, and *Epas1*; down: *Hba-a1*, *Hbb-bs*, and *Hbb-bt*). Taurine is an agonist of GABA receptors and is therefore classified as an inhibitory neurotransmitter.^64^

Diurnal (HWL) mice showed an upregulation of genes associated with glutamate signaling in the habenula, including glutamate receptors and transporters (*Grid1*, *Grik3*, *Grip2*, *Grm3*, *Gclc*, *Slc1a4*, *Slc17a7*, *Glul*, and *Gclm*). Despite the predominantly glutamatergic nature of habenular neurons,^59^ several genes involved in GABAergic signaling showed either upregulation (*Gabbr1*, *Gabbr2*, and *Slc6a1*) or downregulation (*Gabra2*, *Gabarap*, and *Gabrb1*) in diurnal (HWL) mice. The habenula comprises a heterogeneous cell-type distribution,^65,66^ which we confirmed by deconvoluting our bulk murine habenula RNA-seq data: 40% lateral habenula (LHb) neurons, 20% MHb neurons, 30% polydendrocytes, 6% differentiating oligodendrocytes, 3% ependymal cells, and 1% astrocytes/microglia. Interestingly, the expression of the glutamate transporter *Vglut1* (*Slc17a7*) was increased in diurnal mice, while *Vglut2/3* (*Slc17a6* and *Slc17a8*) did not show significant changes. Given that *Vglut1* is exclusively expressed in the MHb and *Vglut2/3* is primarily expressed in the LHb,^59^ it is likely that glutamatergic synaptic neurotransmission in the MHb is affected in diurnal (HWL) mice. Furthermore, the elevated expression of choline acetyl-transferase (*Chat*) in diurnal (HWL) mice suggests the involvement of the MHb, as neurons in this region are primarily cholinergic.^66^ The serotonin receptor (*Htr2c*) was upregulated at the end of the dark phase. Dopamine receptor expression (*Drd2*) was downregulated at ZT17 in diurnal (HWL) mice. Numerous other neurotransmitters, neuropeptides, receptors, and channels in the habenula also exhibited altered expression patterns (Tables S8 and S12). Collectively, these findings indicate that neurotransmitter signaling is modulated in the habenula of diurnal mice, potentially suggesting distinct neuronal activity patterns in the habenula following the nocturnal-diurnal switch.

To identify whether the habenula clock undergoes changes in response to the switch between nocturnal and diurnal behavior, we assessed the phase of clock genes. These transcripts phase advanced with several hours in diurnal mice (Figure 7C). In nocturnal (LWL) mice, rhythmic gene expression in the habenula was synchronized and peaked at ZT6 (light) and ZT17 (dark), involving 1,000 genes (Figures 7D and 7E). Diurnal (HWL) mice exhibited a distinct set of rhythmic genes (768 genes) with prominent peaks occurring at opposite times of the day (ZT10 and ZT22). Among the common rhythmic genes, 45 of 54 displayed a phase advance in diurnal (HWL) mice, with an average phase shift of −4.1 ± 0.3 h (Figures 7F and 7G). Several rhythmic transcripts that underwent phase shifts in the habenula encoded proteins involved in glutamate signaling (*Gpr179*), lipid droplet coating (*Pln4*), hypoxia (*Hyou1*, *P4hb*, and *Plat*), and response to starvation (*Eif2s1*, *Xbp1*, and *Mfsd2a*). Sphingosine kinase 2 (*Sphk2*), which catalyzes sphingosine phosphorylation, and heat-shock proteins (*Hspb1* and *Serpinh1*) were also phase advanced in the habenula (Figure 7H). Furthermore, *Pmp22*, a marker for Schwann cell myelination, was downregulated in the habenula and prefrontal cortex of diurnal (HWL) mice. These findings suggest potential axonal degeneration in energy-deficient mice. Several other genes in the habenula of diurnal (HWL) mice showed phase advances exceeding 5 h, including threonyl-tRNA synthetase-like 2 (*Tarsl2*), proline-rich 13 (*Prr13*), tetraspanin (*Tspan31*), and uridine monophosphate synthetase (*Umps*). Together, the sensitivity of both core clock genes and other rhythmic genes in the habenula to temporal niche switching suggests the potential involvement of neuronal populations in the habenula in the nocturnal-diurnal switch.

## DISCUSSION

Although plasticity in the daily timing of activity has been observed across various species,^5^ little is known about the mechanisms and brain networks involved, and 24-h transcriptome rhythms in brain sites of niche-switched animals have been rarely studied. To address this gap, we implemented the work-for-food model, wherein we manipulated energy balance to switch mice between nocturnality and diurnality, and found distinct gene expression signatures in different brain structures of these mice.

While the SCN showed minimal changes in clock genes, temporal-niche-specific rhythmic genes and phase changes in common rhythmic genes were observed, indicating the sensitivity of the SCN to temporal niche switching. Previous research confirmed a functional role for the SCN in nocturnal-diurnal switches, as negative energy balance failed to increase diurnal activity in SCN-lesioned mice (S.J. Riede and R.A.H., unpublished data). We hypothesize that the SCN clock remains synchronized with the light-dark cycle during niche switching, while another set of rhythmic SCN genes are synchronized to sleep-wake, feeding-fasting, and body temperature cycles, conveying the animal’s current internal state. Unlike the SCN, the molecular clock of extra-SCN regions adapted to the temporal niche switch, which is in line with previous findings showing that timed feeding can entrain central clock rhythms in the HIP and PVN.^67^ It has been well documented that timed feeding can entrain peripheral clock rhythms independent of the SCN.^24,68–90^ There are similarities between timed feeding during daytime and the work-for-food paradigm, since they both do not depend on the SCN to shift its phase. Furthermore, clock-gene rhythms in the SCN also remain unaffected by timed exercise, while timed exercise, similar to timed feeding, acts as a robust zeitgeber for entraining peripheral clocks.^91^ Mice experiencing negative energy balance showed fragmented activity patterns, indicating that they distributed their food intake across the 24-h cycle and dampened their rhythms in behavioral activity, potentially contributing to the reduced amplitude of gene expression, similar to observations in mice on an *ad libitum* high-fat diet.^24,87–89,92–94^ However, it is important to note that the changes in gene expression may not be causal to the behavioral shift, as they could be a consequence of the shift in behavioral activity, as our data cannot differentiate between the causes and the consequences of changes in gene expression.

Hypothalamic brain regions receiving signals about nutrient availability from tanycytes, such as the ARC, DMH, and VMH,^95–97^ surprisingly exhibited minimal gene expression changes independent of time of day. Interestingly, there is evidence indicating the presence of tanycytes in the ependymal cells in the MHb.^63^ The pronounced changes in (clock) gene expression timing and magnitude observed in the habenula matching the nocturnal-diurnal switch indicate its potential role in driving temporal-niche switches. Interestingly, previous studies have shown that disruption of the habenula or its efferent pathways induces hyperactivity.^98–102^ We hypothesize that this brain region potentially serves as an integrator of metabolic status and external time cues to adjust locomotion patterns accordingly (Figure 7I). This integration allows the organism to efficiently regulate physiological processes by combining information from two sources: first, afferent projections from both the SCN^103–106^ and the (peri-)habenula, both receiving signals from the retina,^107–109^ provide external light-dark cycle information to the LHb. Second, by monitoring CSF composition in the third ventricle, which contains vital nutrients, the organism can sense its internal metabolic state and adjust gene expression in the MHb accordingly. Indeed, the MHb and LHb gene expression patterns are very different, suggesting different functions.^65,66^ While our current analysis utilizes bulk RNA-seq data containing both regions, future experiments should incorporate single-cell and spatial transcriptomics to gain mechanistic insights, enabling a more detailed investigation of cell-type-specific clocks within the habenula. Interestingly, the LHb and MHb oscillators run out of phase under conditions of constant darkness or when the SCN is ablated.^110^ We speculate that the uncoupling of these two habenula oscillators may also be initiated by energy deficiency. The integration of signals from the LHb and MHb may then result in a switch in rhythmic gene expression and its downstream nuclei to adjust behavior accordingly during periods of limited energy availability. The habenula plays a crucial role in modulating the activity of the monoaminergic system in the BRS, particularly the dopaminergic (DA) and serotonergic (5-HT) neurons, in turn influencing locomotor behavior.^59^ The proposed mechanism allows the organism to synchronize its internal processes, including sleep-wake cycles and hormone secretion, with the external world, while taking into account its metabolic status and optimizing its energy-saving strategy.

Our findings reveal a role for the habenula in the regulation of energy metabolism and circadian rhythms. Interestingly, dysfunction of the habenula has been linked to various neuropsychiatric disorders.^59^ Anorexia nervosa has shown a higher incidence of depression and sleep disorders.^111^ Moreover, post-anorexia patients showed prolonged changes in habenular connectivity.^112^ Future investigations are required to explore the interaction between energy deficiency, depression, the circadian system, and the involvement of the habenula.

The diurnal-nocturnal transcriptome atlas of the mouse brain generated in our study represents a valuable resource that can contribute to elucidating the underlying mechanisms and brain networks involved in the modulation of the circadian system by metabolic feedback. By shedding light on the molecular pathways through which metabolic signals influence the daily timing of activity relative to the light-dark cycle, our findings hold significant implications for various aspects of human health, including sleep regulation, shift work, chronotherapy, chronomedicine, and metabolic health. The diurnal-nocturnal transcriptome atlas of the mouse brain generated in this study serves as a valuable resource for further research aimed at unraveling the mechanisms and brain networks responsible for the regulation of the circadian system and behavioral rhythms through metabolic feedback.

### Limitations of the study

A major limitation of our study was that the *ad libitum*-fed male CBA/CaJ control mice used in our experiment were not entirely nocturnal prior to the increase in workload, which has been observed in similar studies.^6^ A possible explanation for this might be that the food pellets used are less palatable than the regular lab chow. This might have caused a reduced food intake, accompanied by a minor shift to diurnality in the control mice. Both male and female mice increase daytime activity under negative energy balance,^17^ but male mice show more daytime activity than female mice. Therefore, we specifically chose to use male mice for our study. However, future studies including female mice are needed to investigate sex differences in circadian plasticity. It is also important to consider the effects of the light-dark cycle on our results, particularly since diurnal mice received more light input. Known light-regulated transcripts of the SCN^113^ were unaffected during the light phase of diurnal mice. However, the influence of light on gene expression cannot be completely ruled out in this study. Conducting studies under constant darkness or a skeleton photoperiod would help eliminate the direct effects of light. Furthermore, our study focused on examining temporal niche switching within the same species. Future studies are needed to investigate whether rhythmic gene expression is similarly affected comparing nocturnal and diurnal species. The induction of a diurnal phenotype in our study was achieved by inducing a negative energy balance. It remains to be investigated whether complex interactions between different environmental factors that can trigger a switch involve similar brain networks. The lengthening/fragmentation of the active period in diurnal mice may have resulted in a shortening or more fragmented sleep pattern, indicating partial sleep deprivation. Indeed, several genes associated with sleep deprivation (*Fabp7*, *Cirbp*, and *Gkn3*) were altered. Given that our study contains bulk RNA-seq data, it is important to consider the heterogeneity of cell types within brain tissues. Previous research has shown that distinct cell types within the SCN possess unique circadian gene expression profiles.^114^ It is reasonable to assume that other brain regions also exhibit heterogeneity in the expression of (clock) genes among specific cell types. Integrating single-cell RNA-seq and spatial information in future studies would provide a more comprehensive understanding of the underlying mechanisms of circadian regulation within different brain regions. Finally, our data cannot establish causality and do not differentiate between the causes and the consequences of gene expression changes.

## STAR★METHODS

### RESOURCE AVAILABILITY

#### Lead contact

Further information and requests for resources and reagents should be directed to and will be fulfilled by the lead contact, Satchidananda Panda (satchin@salk.edu).

#### Materials availability

This study did not generate new unique reagents

#### Data and code availability

- All raw (FASTQ files) and processed (raw counts and normalized counts) bulk RNA-seq data discussed in this publication have been deposited on NCBI’s Gene Expression Omnibus (GEO) and are publicly available as of the date of publication through GEO series accession number GEO: GSE228967. Statistical analysis for differential and rhythmic genes, other parameters of gene expression, and raw data from figures can be found in Tables S1, S2, S3, S4, S5, S6, S7, S8, S9, S10, S11, S12, S13, and S14, which were deposited on Mendeley at Mendeley Data: https://doi.org/10.17632/3g7bdj422p.2 and are publicly available as of the date of publication. This paper also analyzes existing, publicly available data. The accession numbers for the datasets are listed in the key resources table.
- This paper does not report original code.
- Any additional information required to reanalyze the data reported in this paper is available from the lead contact upon request.

### EXPERIMENTAL MODEL AND STUDY PARTICIPANT DETAILS

#### Animals

Male CBA/CaJ mice (JAX strain #000654; melatonin proficient inbred mouse strain with intact retina) were bred inside the animal facility at the University of Groningen, The Netherlands. Mice were individually housed in transparent macrolon cages, 32 cm (L) x 13 cm (W) x 15 cm (H) containing a plastic running wheel (Savic, Kortrijk, Belgium; 14 cm in diameter) and spruce wood bedding material (BK8–15; Safe-lab, Rosenberg, Germany). All mice were housed under a 12h light: 12h dark cycle (60–70 lux at cage level during light phase, peak wavelength = 458 nm and 610 nm), at an ambient temperature of 21±1°C and relative humidity of 55±5%. Water was available *ad libitum* throughout the experiment. All animal experiments were performed in accordance with the guidelines of the local animal welfare body (IvD) conform to Directive 2010/63/EU, and approved by the CCD (Centrale Commissie Dierproeven) of the Netherlands (CCD, license number: AVD1050020186147).

#### “Work-for-food” procedure

12-week old male mice were individually housed in an experimental cage equipped with a food pellet dispenser (ENV-203–45; Med Associates Inc., St. Albans, VT, USA), which was controlled by running wheel activity. A 45-mg grain-based dustless precision food pellet (21% protein, 4% fat, 54% carbohydrate, 630 J per pellet; F0165, BioServ, Flemington, NJ, USA) was delivered via a feeder tube into the animal cage after a set number of wheel revolutions. After 3 weeks of habituation, animals were assigned to either a low workload (LWL) or a high workload (HWL) paradigm. All animals (15-week-old, ~26 grams, n = 24) started at a low workload = 100 revolutions /pellet = 6.8 meter/kiloJoule (m/kJ). For high workload animals, workload was daily increased by 20 revolutions /pellet (1.4 m/kJ) for the first 3 days, thereafter workload was daily increased by 10 revolutions /pellet (0.7 m/kJ), to induce a negative energy balance over time. For low workload animals, workload was maintained around 100 revolutions /pellet (6.8 m/kJ). The low workload group is similar to *ad libitum* condition, because food pellets were always present inside the cage.

Moreover, low workload animals obtained on average 67±1 kJ/day (Figure 1A), which was similar to the daily food intake (~70 kJ/day) in similar age male C57BL/6J mice housed with running wheels.^20,21^ The ‘work-for-food’ paradigm allows animals to run, obtain food, and eat at all times of day. Animals were weighed every two days between ZT10-ZT12, where ZT0 is the time when light is ON, and ZT12 is when light is OFF. An extensive description of the ‘work-for-food’ paradigm in small rodents can be found here.^18^

### METHOD DETAILS

#### Wheel-running behavior

Each mouse’s daily running wheel activity patterns were recorded using a Circadian Activity Monitoring System (CAMS; developed by HM Cooper, INSERM, Bron, France). The activity was recorded continuously in 1-minute bins until tissue collection. Actograms were generated using ActoView for MS Excel 2010 (programmed by C. Mulder, University of Groningen). ChronoShop (version 1.1) was used for the calculation of circadian characteristics of wheel-running activity (i.e. onset, center of gravity (COG) and offset).^116^ To calculate the percentage daily daytime activity (0–100%), the total number of wheel revolutions during the light phase was divided by the total daily number of wheel revolutions over the 24-h day. Baseline activity profiles were calculated by averaging day −4 to 0. Condition (LWL vs. HWL) activity profiles were an average of the last 5 days before tissue collections.

#### Tissue collection and processing

After 3 weeks in the ‘work-for-food’ paradigm, 2 mice per condition were sacrificed by decapitation every 4 hours over a 24-hour period at ZT1, 5, 9, 13, 17 and 21 ±15min. Animals that were sacrificed during the dark phase, were decapitated under low intensity red dim light (< 0.1 lux, peak wavelength = 659 nm). Trunk blood was directly collected and peripheral tissues, brown adipose tissue (BAT), testis (TES), liver (LIV), quadriceps muscle (QUA), were removed and flash-frozen in liquid N_2_ within 10 minutes after decapitation. Whole brains were extracted from the skull, and directly placed into ice-cold (4°C) cutting media (DMEM, pH 7; Sigma-Aldrich, St. Louis, MO, USA). The dura layers were carefully removed, and the frontal cortex (PRC), cerebellum (CER), brainstem (BRS) and bulbus olfactory (OLB) were dissected. Subsequently, the remaining brain tissue was mounted on a metal block by using ethylcyanoacrylate (super glue), and was placed in a temperature-controlled ice batch at 4°C. 500mm coronal brain slices were prepared by using a vibratome (7000smz, Campden Instruments, Loughborough, Leics, UK). Brain areas: preoptic area (POA), suprachiasmatic nuclei (SCN), periventricular zone (PVZ), paraventricular nuclei (PVN), lateral hypothalamus caudal (LH_C_), lateral hypothalamus rostral (LH_r_), paraventricular nucleus of the thalamus (PVT), Habenula (HAB), arcuate Nucleus (ARC), ventromedial hypothalamus (VMH), dorsomedial hypothalamus (DMH), cortex (COR) and hippocampus (HIP) were further dissected in this order under a dissection microscope as illustrated in Figures S1D–S1G. All collected tissues (Figure 2G) were snap-frozen in liquid N_2_ directly after dissection and stored at −80°C until RNA extraction. Tissues were taken and snap-frozen in the same specific order for each mouse, and all tissues were snap-frozen within 34–59 minutes after decapitation.

#### Glucose measurements

Trunk blood was collected after decapitation from a different cohort of LWL and HWL animals (n=67) at 12 different time points throughout the 24-hour LD-cycle (ZT2, 4, 6, 8, 10, 12, 14, 16, 18, 20, 22, 24). Blood was directly centrifuged at 4°C after which plasma was taken. Plasma glucose levels were determined by colorimetric analysis by using the ferry-cyanide method^124^ in a Technicon auto-analyzer.

#### RNA extraction

Total RNA was extracted from neural and peripheral tissues using 0.5 ml TRIzol reagent (Invitrogen, Carlsblad, CA, USA) according to the manufacturer’s instructions, after tissue disruption using a 5mm RNase free stainless-steel bead and a TissueLyser II (Qiagen, Hilden, Germany) (2×2 min at 30 Hz). To increase RNA yield, 1 μl glycoblue coprecipitant (AM9516, Invitrogen) was added to the isopropanol step for small brain sites (ARC, DMH, HAB, LHc, LHr, POA, PVN, PVT, PVZ, SCN, VMH). Extracted RNA was quantified using a nanodrop One spectrophotometer (Thermo Scientific, Waltham, MA, USA).

**Table:** 

Tissue	RNA extraction method	Sequencing platform	Sequencing depth
SCN	TRIzol^™^, glycoblue	Illumina Novaseq 6000	PE150
POA	TRIzol^™^, glycoblue	Illumina Novaseq 6000	PE100
PVN	TRIzol^™^, glycoblue	Illumina Novaseq 6000	PE150
PVZ	TRIzol^™^, glycoblue	Illumina Novaseq 6000	PE100
DMH	TRIzol^™^, glycoblue	Illumina Novaseq 6000	PE150
VMH	TRIzol^™^, glycoblue	Illumina Novaseq 6000	PE100
ARC	TRIzol^™^, glycoblue	Illumina Novaseq 6000	PE100
LHr	TRIzol^™^, glycoblue	Illumina Novaseq 6000	PE100
LHc	TRIzol^™^, glycoblue	Illumina Novaseq 6000	PE150
HAB	TRIzol^™^, glycoblue	Illumina Novaseq 6000	PE100
PVT	TRIzol^™^, glycoblue	Illumina Novaseq 6000	PE100
OLB	TRIzol^™^	Illumina Novaseq 6000	PE100
PRC	TRIzol^™^	Illumina Novaseq 6000	PE100
COR	TRIzol^™^	Illumina Novaseq 6000	PE100
HIP	TRIzol^™^	Illumina Novaseq 6000	PE100
CER	TRIzol^™^	Illumina Novaseq 6000	PE100
BRS	TRIzol^™^	Illumina Novaseq 6000	PE100
BAT	TRIzol^™^	Illumina Novaseq 6000	PE100
LIV	TRIzol^™^	Illumina Novaseq 6000	PE100
TES	TRIzol^™^	Illumina Novaseq 6000	PE100
QUA	TRIzol^™^	Illumina Novaseq 6000	PE100

#### mRNA library preparation and sequencing

Libraries were prepared using Illumina’s TruSeq Stranded mRNA (PolyA+) kit (Illumina, San Diego, CA, USA) according to the manufacturer’s instructions using half volumes. 300 ng of total RNA was used as input to generate libraries. Unique dual (i5 and i7) 8 base pair sequencing indexes for Illumina-TruSeq DNA were used to allow multiplexing of 96 library samples for next-generation sequencing (Illumina). Quality and quantity of libraries were inspected by using a nanodrop spectrophotometer (Thermo Scientific), Quant-IT^™^ dsDNA HS assay kit (ThermoFisher Scientific) and gel electrophoresis. Libraries were pooled and sequencing was performed using NovaSeq 6000 (Illumina) to obtain paired-end 100 or 150 base pair reads with a target sequencing depth of 25 million reads per sample.

#### Read mapping, annotation, quantification, and normalization

Sequencing quality was assessed using FastQC, version 0.11.9 (Babraham Bioinformatics, Cambridge, UK). 492 samples passed quality control, and an overview of the samples that were included in the analysis can be found in Table S5. Cutadapt was used to trim adapter sequences.^117^ Sequence reads were aligned to the *Mus musculus* (house mouse) genome GRCm38 (mm10) and counted per gene using STAR aligner.^118^ Sequencing depth was an average of 25M uniquely mapped reads (87%) per sample (Table S5). One sample (TES_L_ZT21_X27) was defined as an outlier and was removed from the downstream analysis. Gene counts were filtered for low counts and normalized per tissue using the DESeq2 workflow (Love et al., 2014).

#### Differential gene expression analysis

Differential gene expression analysis per tissue was carried out using DESeq2,^119^ with a design that accounted for workload (LWL, HWL), and LWL was set as a reference level for the analysis. The nbinomWaldTest was used for statistical testing. Subsequently, a variance stabilizing transformation (VST) was used to transform the gene-level count data for visualization.^125^ Principal component analysis (PCA) plots were generated using DESeq2 for all brain or peripheral tissues combined (Figure 2H), all hypothalamic tissues combined (Figure S3), and for each tissue separately (Figure S4). Genes were considered expressed if normalized average gene counts were ≥10.On average, 16,323 genes were expressed per tissue (Table S4). Differentially expressed (DE) genes were defined as Benjamini and Hochberg adjusted p < 0.05. Statistics of differential gene expression analysis can be found in Table S8.

#### Rhythmic gene expression analysis

To detect rhythmic signals from our time-series gene expression data, the function meta2d, within the MetaCycle R package was used on the normalized counts.^120^ Genes were considered rhythmically expressed if integrated JTK_CYCLE and Lomb-Scargle was p < 0.05. Amplitude and phase were derived from meta2d_AMP and meta2d_phase respectively. CircaCompare was used to test for significant phase shifts and amplitude changes in rhythmically expressed genes.^126^ Statistics of rhythmic gene expression analysis can be found in Table S1.

#### Mitochondrial and ion channel genes

Output gene lists from rhythmic and DE genes analysis were compared to several gene lists: MitoCarta 3.0 is a gene list of 1140 mouse mitochondrial genes was used.^127^ 2,920 genes that encode for ion channels were retrieved from https://www.guidetopharmacology.org/GRAC/IonChannelListForward?class=VGIC (Tables S12 and S13).

#### Gene ontology

Gene ontology (GO) analysis was performed on groups of differential expressed genes and cycling genes using the gene annotation tool Metascape.^121^ Top 6 most significant non-redundant GO terms were used (Table S3).

#### Bulk tissue cell type deconvolution

Cell type composition of the habenula used for bulk RNA-seq was characterized by deconvolution with single-cell RNA-seq datasets for the habenula,^65,66^ using the MuSiC package.^122^

#### Baboon data set

For comparing between mouse (*Mus musculus*) and the primate baboon (*Papio anubis*) time-series gene expression, RNA-seq data from 14 different tissues (ARC, CER, DMH, HAB, HIP, LH, LIV, OLB, POA, PRC, PVN, SCN, TES, VMH) were used from a previously published study.^28^ Mouse and baboon data were normalized between 0 and 1 for comparison.

### QUANTIFICATION AND STATISTICAL ANALYSIS

#### Statistical analysis

One-way repeated measures ANOVA was used when analyzing the effect of workload within experimental groups. One-way ANOVA was used to analyze the effect of workload on activity parameters between experimental groups. All analysis and statistical tests were performed in RStudio (version 2022.07.1; http://www.rstudio.com/). Unless otherwise described, all data are presented as mean ± SEM, with *p < 0.05, **p < 0.01, ***p < 0.001. All figures were generated using the R-package ‘ggplot2’.^124^

## Supplementary Material

1

2

3

4

5

6

7

9

10

11

12

13

14

15

16

## Figures and Tables

**Table:** KEY RESOURCES TABLE

REAGENT or RESOURCE	SOURCE	IDENTIFIER
Critical commercial assays		
DMEM Cutting media, pH 7	Sigma-Aldrich	D2902
TRIzol^™^ Reagent	Invitrogen	15596026
Quant-IT^™^ DNA Assay Kit	Thermo Scientific	Q33120
TruSeq Stranded mRNA kit	Illumina	20020595

Deposited data		

Raw files for RNA-seq	This paper	GEO: GSE228967
Raw counts, normalized counts	This paper	GEO: GSE228967
Statistical analysis for differential and rhythmic gene expression	This paper; Mendeley data	Tables S1, S2, S3, S4, S5, S6, S7, S8, S9, S10, S11, S12, S13, and S14; Mendeley Data: https://doi.org/10.17632/3g7bdj422p.2
RNA-seq dataset, baboon tissues	Mure et al.^28^	GEO: GSE98965

Experimental models: Organisms/strains		

Mouse: CBA/CaJ	The Jackson Laboratory	JAX:000654

Software and algorithms		

MedPC IV	Med Associates	https://med-associates.com/product/med-pc-v/
Circadian Activity Monitoring System (CAMS)	Developed by HM Cooper, INSERM, Bron, France	N/A
R, v4.1.2	R Development Core Team, 2022	https://www.r-project.org
RStudio, v2022.07.1+554	RStudio: Integrated Development for R, Boston, MA	https://www.rstudio.com
ActoView for MS Excel 2010	Developed by C. Mulder, University of Groningen	N/A
ChronoShop, v1.1	Spoelstra et al.^116^	https://dataverse.nl/dataset.xhtml?persistentId=hdl:10411/YHJEFV
Cutadapt, v4.1	Martin etal.^117^	https://cutadapt.readthedocs.io/en/stable/
FastQC, v0.11.9	Babraham Bioinformatics, Cambridge, UK	https://www.bioinformatics.babraham.ac.uk/projects/fastqc/
STAR, v2.7.10a	Dobin et al.^118^	https://github.com/alexdobin/STAR
DESeq2, v1.34.0	Love et al.^119^	https://bioconductor.org/packages/release/bioc/html/DESeq2.html
Metacycle, v1.2.0	Wuetal.^120^	https://github.com/gangwug/MetaCycle
Metascape, v3.5.20230101	Zhou et al.^121^	https://metascape.org/gp/index.html#/main/step1
MuSiC, v0.2.0	Wang et al.^122^	https://github.com/xuranw/MuSiC
Ggplot2, v3.3.5	Wickham et al.^123^	https://ggplot2.tidyverse.org

Other		

45-mg grain-based dustless precision food pellets	BioServ	F0165
Automated feeders	Med Associates	ENV-203-45

## Highlights

- The SCN clock is resistant to a behavioral nocturnal-diurnal switch
- Extra-SCN clocks adapt to a behavioral nocturnal-diurnal switch
- Nocturnal and diurnal phenotypes are associated with specific sets of rhythmic genes
- The habenula is most affected, implying a role in driving nocturnal-diurnal switches
