# Supplementary material for: Energy balance drives diurnal and nocturnal brain transcriptome rhythms
Source: Cell Rep. Author manuscript; Available in PMC 2024 Aug 18. (PMC11330649; doi:10.1016/j.celrep.2024.113951)
Supplement: 1 [file NIHMS1980970-supplement-1.pdf]

Cell Reports, Volume *43*

Supplemental information

Energy balance drives diurnal and nocturnal brain transcriptome rhythms

Laura van Rosmalen, Shaunak Deota, Geraldine Maier, Hiep D. Le, Terry Lin, Ramesh K. Ramasamy, Roelof A. Hut, and Satchidananda Panda

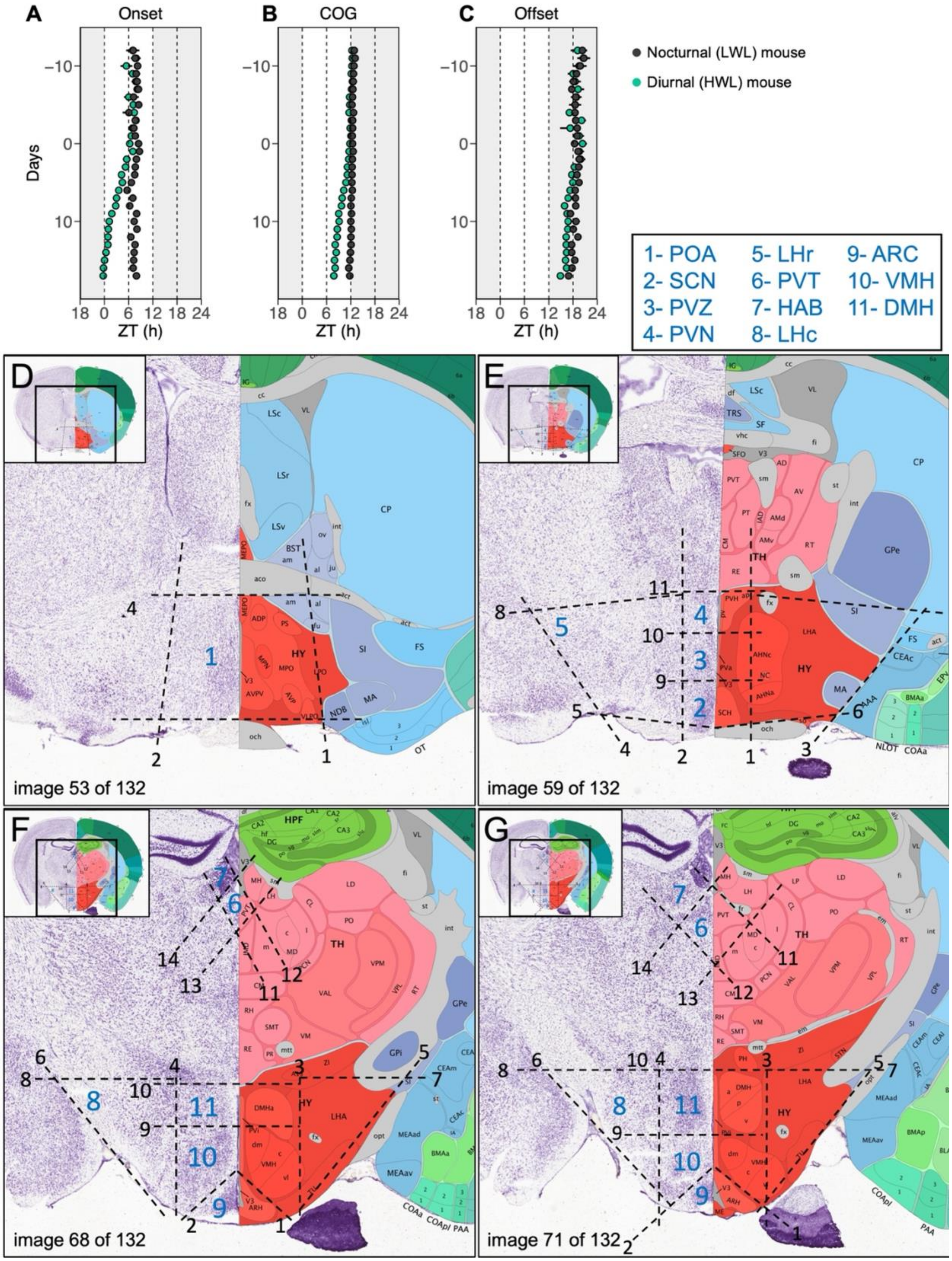

A
Onset
B
COG
C
Offset
Days
ZT (h)
Nocturnal (LWL) mouse
Diurnal (HWL) mouse
1- POA
2- SCN
3- PVZ
4- PVN
5- LHr
6- PVT
7- HAB
8- LHc
9- ARC
10- VMH
11- DMH
D
image 53 of 132
E
image 59 of 132
F
image 68 of 132
G
image 71 of 132
HY
TH
HPF

Supplementary figure 1. Changes in activity patterns in low workload and high workload mice and dissections of specific brain regions from 500 μm coronal brain slices. Related to Figures 1, 2 and STAR Methods. (A) Onset, (B) center of gravity (COG), and (C) offset of activity in low workload (black) and high workload (green) mice. Data are plotted as mean ± SEM. Day 0 represents the start of the experiment. Shaded areas represent the dark phase. (D-G) Cuts are made with a scalpel in the specified order, depicted as dashed lines. Dissected brain regions are annotated in blue: 1 = POA; 2 = SCN; 3 = PVZ; 4 = PVN; 5 = LHR; 6 = PVT; 7 = HAB; 8 = LHC; 9 = ARC; 10=VMH; 11 = DMH. Nissl (left) and anatomical annotations (right) from the Allen Mouse Brain Atlas and Allen Reference Atlas – Mouse Brain, mouse.brain-map.org and atlas.brain-map.org.

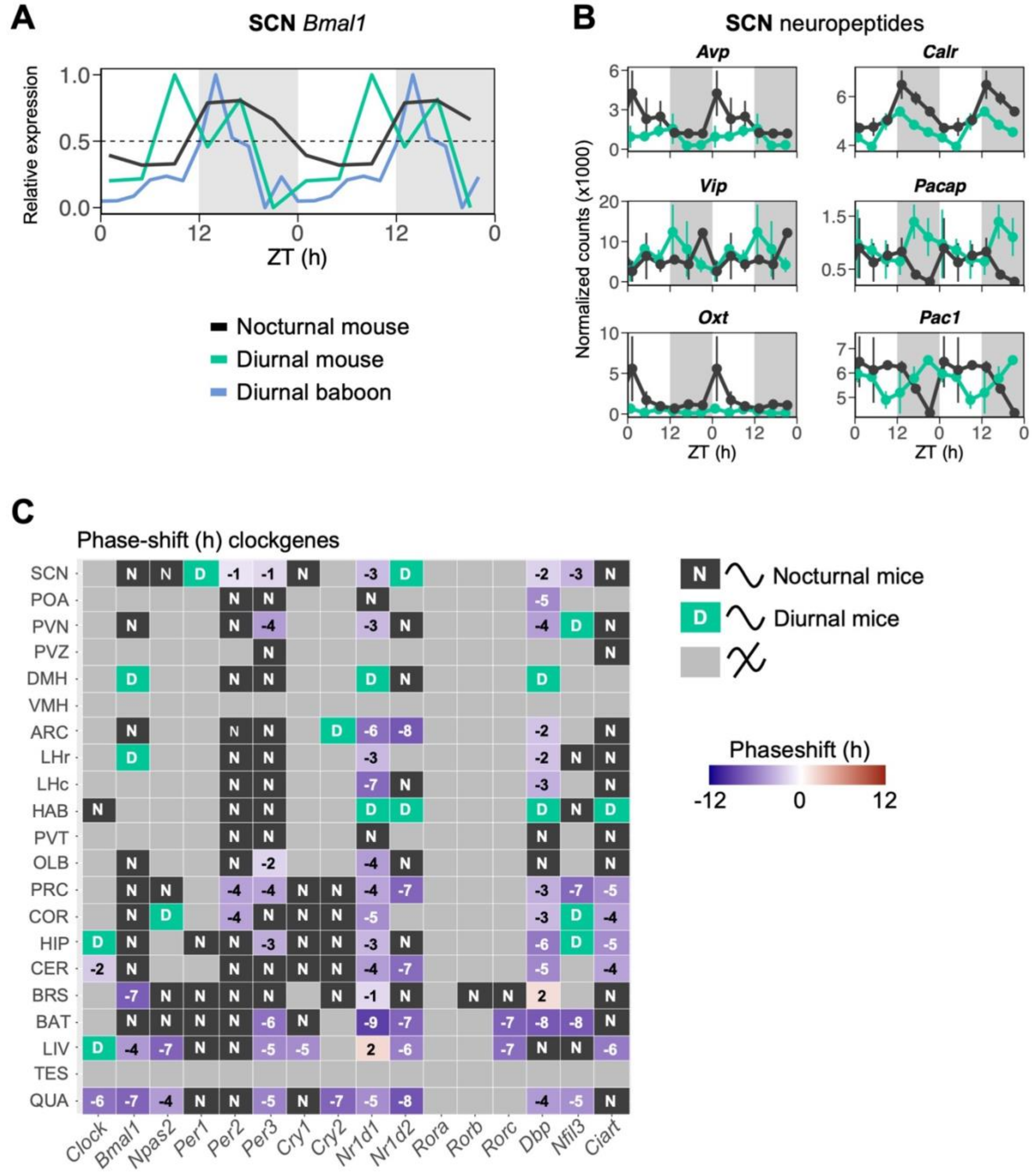

Supplementary figure 2. Clock gene and neuropeptide expression in nocturnal and diurnal mice. Related to Figures 2 and 3. Double-plotted relative expression profiles of clock gene (A) *Bmal1* and (B) SCN neuropeptides**.** Shaded areas represent the dark phase. (C) Heatmap showing the phase change in the peak phase (in hours) of clock gene expression across tissues that were significantly cycling in nocturnal and diurnal mice. Genes detected as rhythmic in only nocturnal or diurnal mice are indicated in black and green respectively. Genes that were not cycling in a specific tissue are indicated in grey.

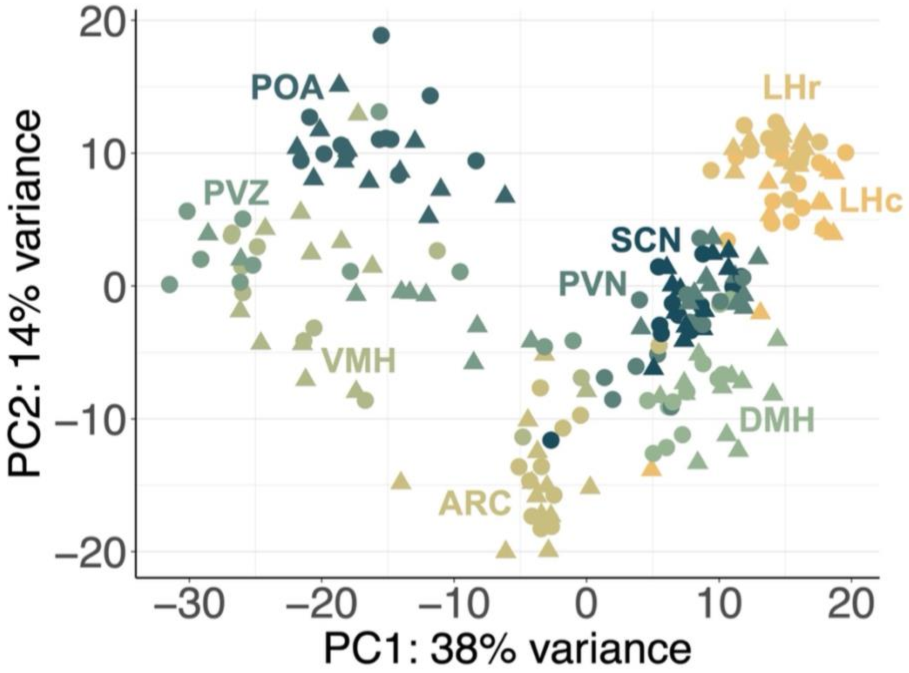

Supplementary figure 3. Principal component analysis performed on gene expression levels of hypothalamic sites. Related to Figure 2.

Circles indicate low workload mice; triangles indicate high workload mice.

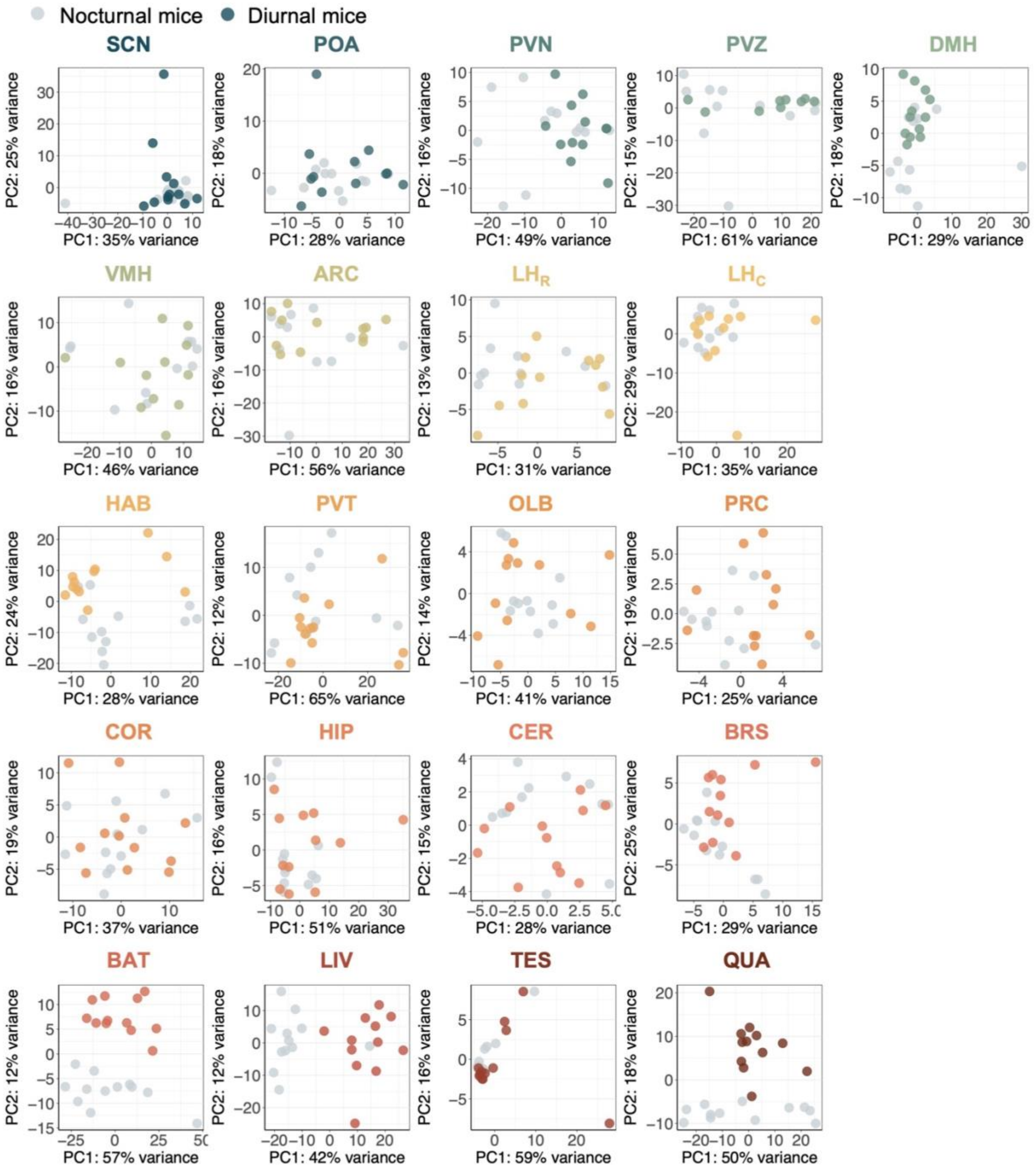

Supplementary figure 4. PCA plots based on gene expression level per tissue. Related to Figure 2.

Grey circles indicate low workload mice; colored circles indicate high workload mice. SCN, suprachiasmatic nucleus; POA, preoptic area; PVN, paraventricular nuclei; PVZ, periventricular zone; DMH, dorsomedial hypothalamus; VMH, ventromedial hypothalamus; ARC, arcuate nucleus; LH_R, lateral hypothalamus rostral; LH_C, lateral hypothalamus caudal; HAB, habenula; PVT, paraventricular nucleus of the thalamus; OLB, olfactory bulb; PRC, prefrontal cortex; COR, cortex; HIP, hippocampus; CER, cerebellum; BRS, brainstem; BAT, brown adipose tissue; LIV, liver; TES, testis; QUA, Quadriceps.

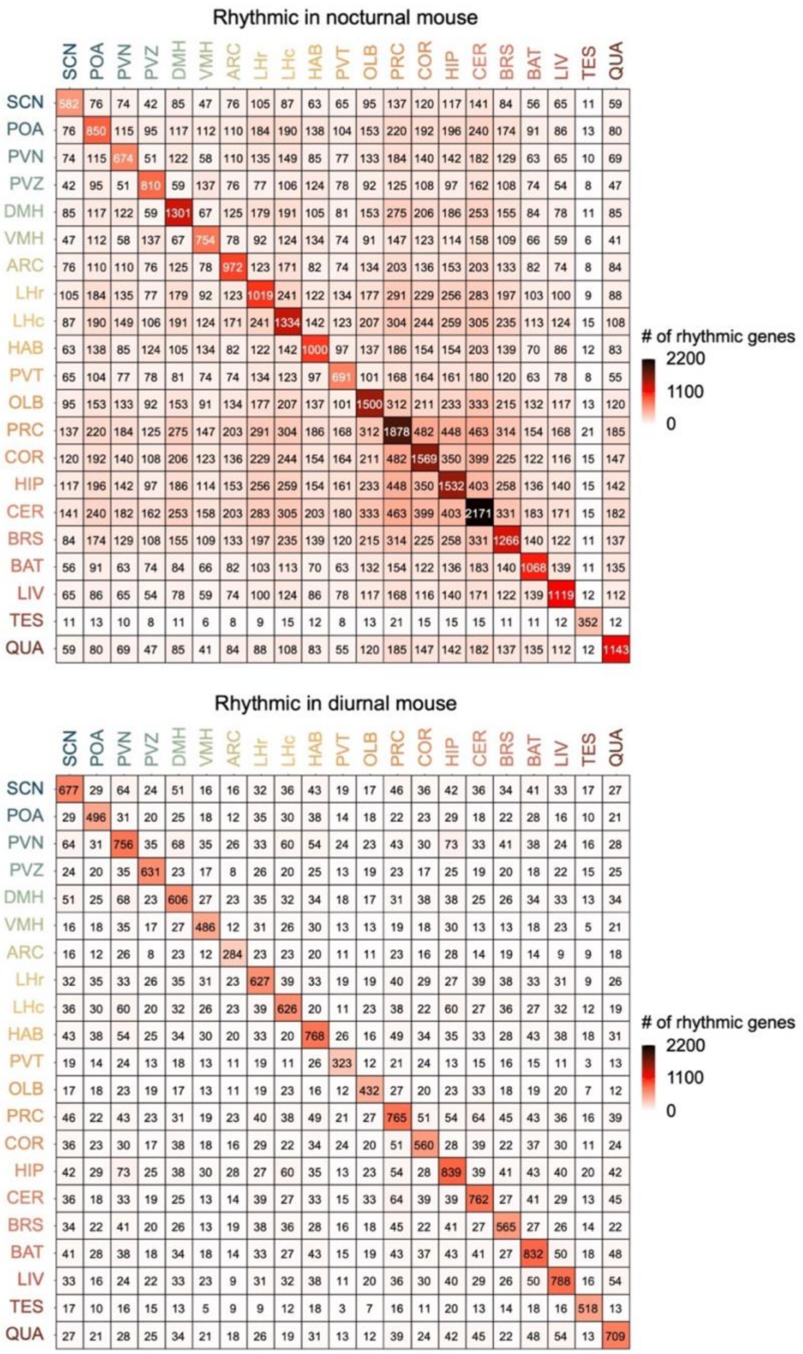

Supplementary figure 5. Tissue by tissue overlap of rhythmic genes in each tissue in nocturnal mice and diurnal mice. Related to Figure 4.

SCN, suprachiasmatic nucleus; POA, preoptic area; PVN, paraventricular nuclei; PVZ, periventricular zone; DMH, dorsomedial hypothalamus; VMH, ventromedial hypothalamus; ARC, arcuate nucleus; LH_R, lateral hypothalamus rostral; LH_C, lateral hypothalamus caudal; HAB, habenula; PVT, paraventricular nucleus of the thalamus; OLB, olfactory bulb; PRC, prefrontal cortex; COR, cortex; HIP, hippocampus; CER, cerebellum; BRS, brainstem; BAT, brown adipose tissue; LIV, liver; TES, testis; QUA, Quadriceps. See also Table S14.

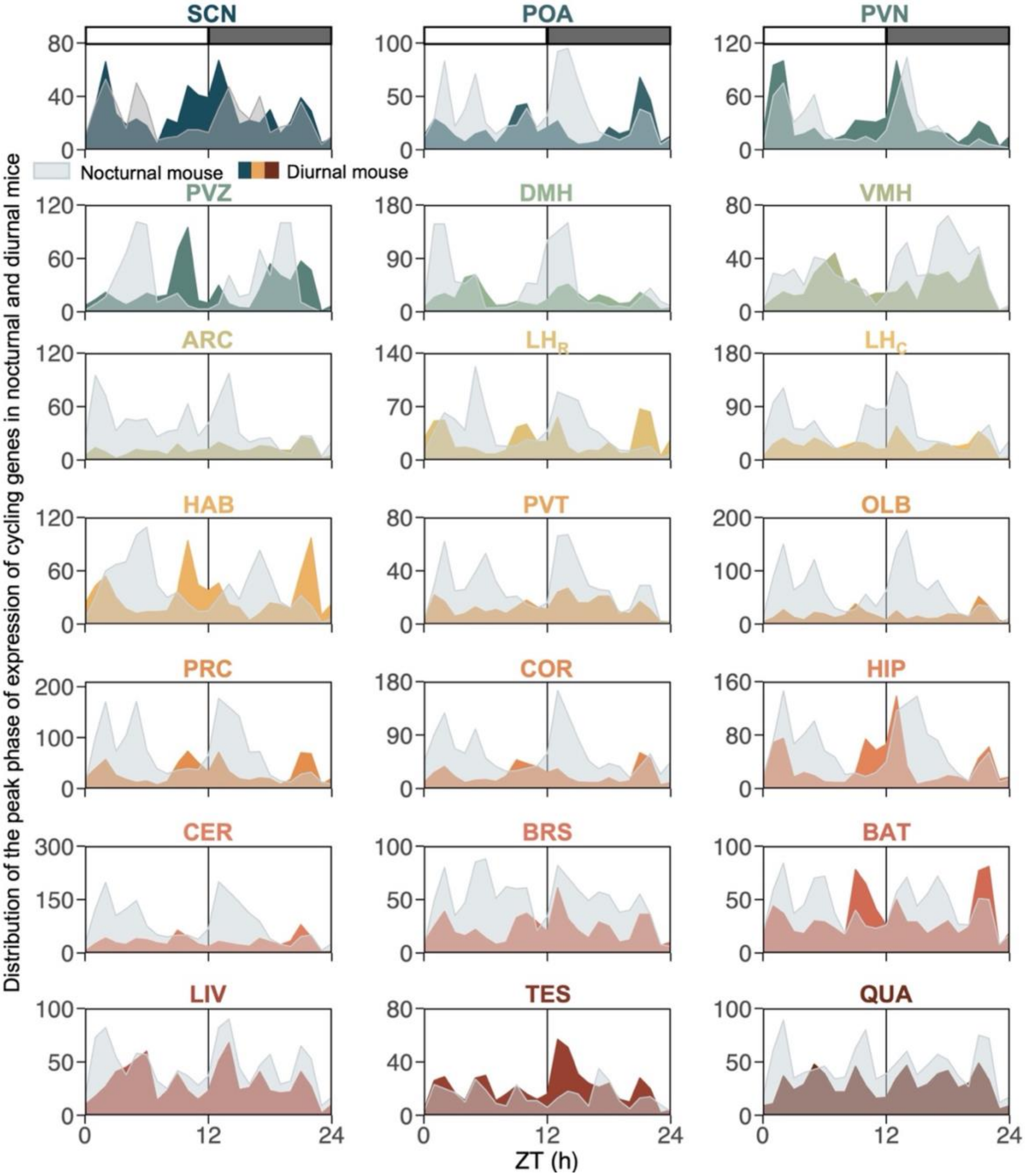

Supplementary figure 6. Phase of rhythmic gene expression changes in diurnal mice in a tissue-specific manner. Related to Figure 4.

Temporal distribution of peak phases of expression of cycling genes in nocturnal (grey) and diurnal (colors) mice in different tissues. Y-axis indicates the number of cycling genes that peak in expression at ZT0-ZT24 (1-hour bins). The grey bar on top indicates lights OFF. SCN, suprachiasmatic nucleus; POA, preoptic area; PVN, paraventricular nuclei; PVZ, periventricular zone; DMH, dorsomedial hypothalamus; VMH, ventromedial hypothalamus; ARC, arcuate nucleus; LH_R, lateral hypothalamus rostral; LH_C, lateral hypothalamus caudal; HAB, habenula; PVT, paraventricular nucleus of the thalamus; OLB, olfactory bulb; PRC, prefrontal cortex; COR, cortex; HIP, hippocampus; CER, cerebellum; BRS, brainstem; BAT, brown adipose tissue; LIV, liver; TES, testis; QUA, Quadriceps.

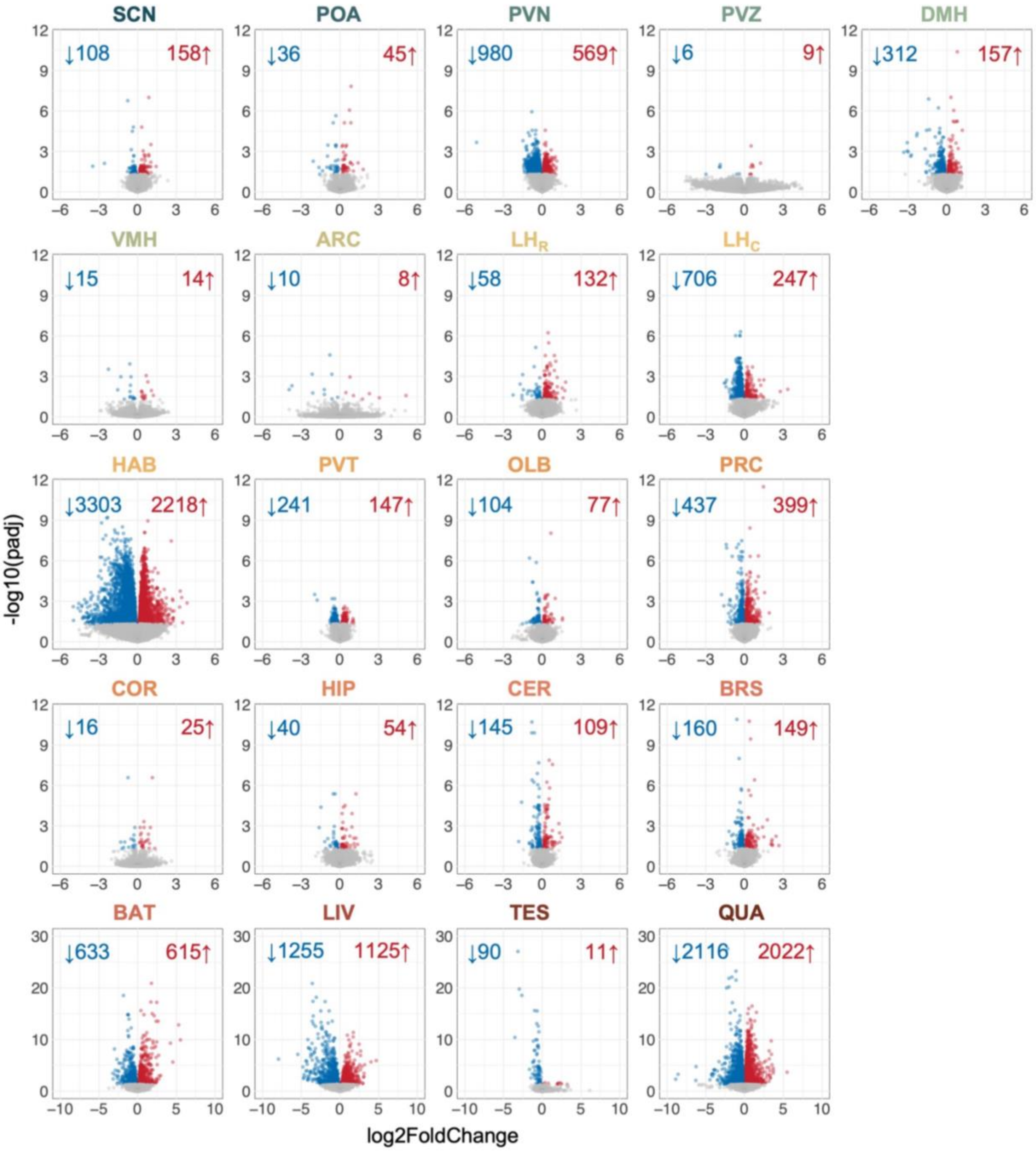

Supplementary figure 7. Volcano plots of differentially expressed genes per tissue. Related to Figure 6.

Genes that are upregulated in diurnal mice are depicted in red, genes that are downregulated in diurnal mice are depicted in blue. SCN, suprachiasmatic nucleus; POA, preoptic area; PVN, paraventricular nuclei; PVZ, periventricular zone; DMH, dorsomedial hypothalamus; VMH, ventromedial hypothalamus; ARC, arcuate nucleus; LH$_R$, lateral hypothalamus rostral; LH$_C$, lateral hypothalamus caudal; HAB, habenula; PVT, paraventricular nucleus of the thalamus; OLB, olfactory bulb; PRC, prefrontal cortex; COR, cortex; HIP, hippocampus; CER, cerebellum; BRS, brainstem; BAT, brown adipose tissue; LIV, liver; TES, testis; QUA, Quadriceps.

Table S1: Metacycle statistics
Table S2: List of cycling genes
Table S3: List of GO enrichment terms
Table S4: No of genes expressed
Table S5: No of reads
Table S6: Phases of the core clock genes across tissues
Table S7: Genes cycling in tissues
Table S8: Statistical analysis of differentially expressed genes (DE) in each tissue
Table S9: List of differentially expressed genes in each tissue padj<0.05
Table S10: Genes differentially expressed in tissues
Table S11: Tissue overlap of differentially expressed genes
Table S12: Lists of differentially expressed ion channels
Table S13: Lists of cycling mitochondrial genes
Table S14: Tissue overlap cycling genes

nocturnal_diurnal_mouse_tissue_expression_raw_counts.csv
nocturnal_diurnal_mouse_tissue_expression_normalized_counts.csv

References

Allen Institute for Brain Science (2004). Allen Mouse Brain Atlas [dataset]. Available from mouse.brain-map.org.Allen Institute for Brain Science (2011).
Allen Reference Atlas – Mouse Brain [brain atlas]. Available from atlas.brain-map.org.
